# Pericyte dysfunction and loss of interpericyte tunneling nanotubes promote neurovascular deficits in glaucoma

**DOI:** 10.1073/pnas.2110329119

**Published:** 2022-02-08

**Authors:** Luis Alarcon-Martinez, Yukihiro Shiga, Deborah Villafranca-Baughman, Nicolas Belforte, Heberto Quintero, Florence Dotigny, Jorge L. Cueva Vargas, Adriana Di Polo

**Affiliations:** ^a^Department of Neuroscience, Université de Montréal, Montréal, QC H3C 3J7, Canada;; ^b^Neuroscience Division, Centre de Recherche du Centre Hospitalier de l’Université de Montréal, Montréal, QC H2X 0A9, Canada;

**Keywords:** neurovascular coupling, pericytes, retina, glaucoma, calcium homeostasis

## Abstract

Reduced blood flow and impaired neurovascular coupling are recognized features of glaucoma, the leading cause of irreversible blindness worldwide, but the mechanisms underlying these defects are unknown. Retinal pericytes regulate microcirculatory blood flow and coordinate neurovascular coupling through interpericyte tunneling nanotubes (IP-TNTs). Using two-photon microscope live imaging of the mouse retina, we found reduced capillary diameter and impaired blood flow at pericyte locations in eyes with high intraocular pressure, the most important risk factor to develop glaucoma. We show that IP-TNTs are structurally and functionally damaged by ocular hypertension, a response that disrupted light-evoked neurovascular coupling. Pericyte-specific inhibition of excessive Ca^2+^ influx rescued hemodynamic responses, protected IP-TNTs and neurovascular coupling, and enhanced retinal neuronal function as well as survival in glaucomatous retinas. Our study identifies pericytes and IP-TNTs as potential therapeutic targets to counter ocular pressure-related microvascular deficits, and provides preclinical proof of concept that strategies aimed to restore intrapericyte calcium homeostasis rescue autoregulatory blood flow and prevent neuronal dysfunction.

## Significance

The current lack of understanding of the mechanisms leading to neurovascular deficits in glaucoma is a major knowledge gap in the field. Retinal pericytes regulate microcirculatory blood flow and coordinate neurovascular coupling through interpericyte tunneling nanotubes (IP-TNTs). We demonstrate that pericytes constrict capillaries in a calcium-dependent manner during glaucomatous stress, decreasing blood supply and compromising neuronal function. Moreover, ocular hypertension damages IP-TNTs and impairs light-evoked neurovascular responses. The reestablishment of calcium homeostasis in pericytes restores vascular and neuronal function, and prevents retinal ganglion cell death in glaucomatous eyes. This study provides important insights into the therapeutic potential of pericytes to counter vascular dysregulation in glaucoma.

Glaucoma is the leading cause of irreversible blindness worldwide, affecting 80 million people globally in 2020 (1). There is no cure for glaucoma and current therapies rely solely on controlling high intraocular pressure, the major risk factor for developing the disease, albeit with limited success. A crucial element in the pathophysiology of glaucoma is the gradual loss of retinal ganglion cells (RGCs), neurons with long projecting axons that form the optic nerve and establish terminals in the brain. RGCs are metabolically active, and thus require precise regulation of blood supply to meet their oxygen and nutrient demand (2). The vascular theory of glaucoma proposes that insufficient blood flow contributes to RGC neurodegeneration (3). Glaucoma patients suffer from vascular deficits that include decreased blood flow in the retina and optic nerve, reduced vessel caliber, and capillary defects (4–8). Notably, vascular autoregulation and flicker-induced neurovascular coupling, a key process required to match blood flow to the metabolic demand of active neurons, are severely compromised in this disease (9–12). Notwithstanding, the cellular mechanisms underlying vascular dysfunction in glaucoma and their impact on neuronal damage are currently unknown.

Pericytes, the ensheathing cells that wrap around capillary walls, have emerged as key regulators of microcirculatory blood flow and neurovascular coupling (13–17). Pericytes are centrally positioned within the neurovascular unit, contain contractile proteins, and respond rapidly to neuronal stimulation (18, 19). Despite their critical role, pericytes have been understudied in the context of neurodegenerative diseases. The retinal microvasculature is rich in pericytes, with >90% pericyte coverage in human retinal capillaries (20). Location-dependent heterogeneity among pericytes has been recognized in several systems (21). However, capillary pericytes in the retina express contractile proteins, notably α-smooth muscle actin protein (α-SMA) encoded by the *Acta2* gene, independent of whether they are located on superficial or deep vascular plexuses (18, 22). Furthermore, retinal pericytes across vascular beds share the ability to change capillary diameter and modify blood flow (15, 18). The recent discovery of interpericyte tunneling nanotubes (IP-TNTs), fine tubular processes that connect retinal pericytes on distal capillary systems, sheds new light on how blood is distributed within retinal capillary networks in response to neuronal activity (15). In the retina, IP-TNTs are abundant in all vascular plexuses and play an essential role in pericyte-to-pericyte communication and neurovascular coupling (15). Despite this, the role of pericytes and IP-TNTs in vascular dysregulation in glaucoma is unknown . Here, we used two-photon microscopy live imaging in a preclinical model of ocular hypertension (OHT) glaucoma to ask the following questions: 1) Do alterations in pericytes and IP-TNTs drive microvascular deficits? 2) What are the molecular mechanisms underlying pericyte dysfunction? 3) Do pericyte-mediated vascular defects directly affect RGC function? Our data support a crucial role for pericytes and IP-TNTs in microvascular deficits and neurovascular coupling impairment in glaucoma, and provide insights into the mechanisms underlying pericyte and IP-TNT dysfunction.

## Results

### Pericyte-Dependent Microvascular Dysfunction in Glaucoma.

**Fig. 1.**
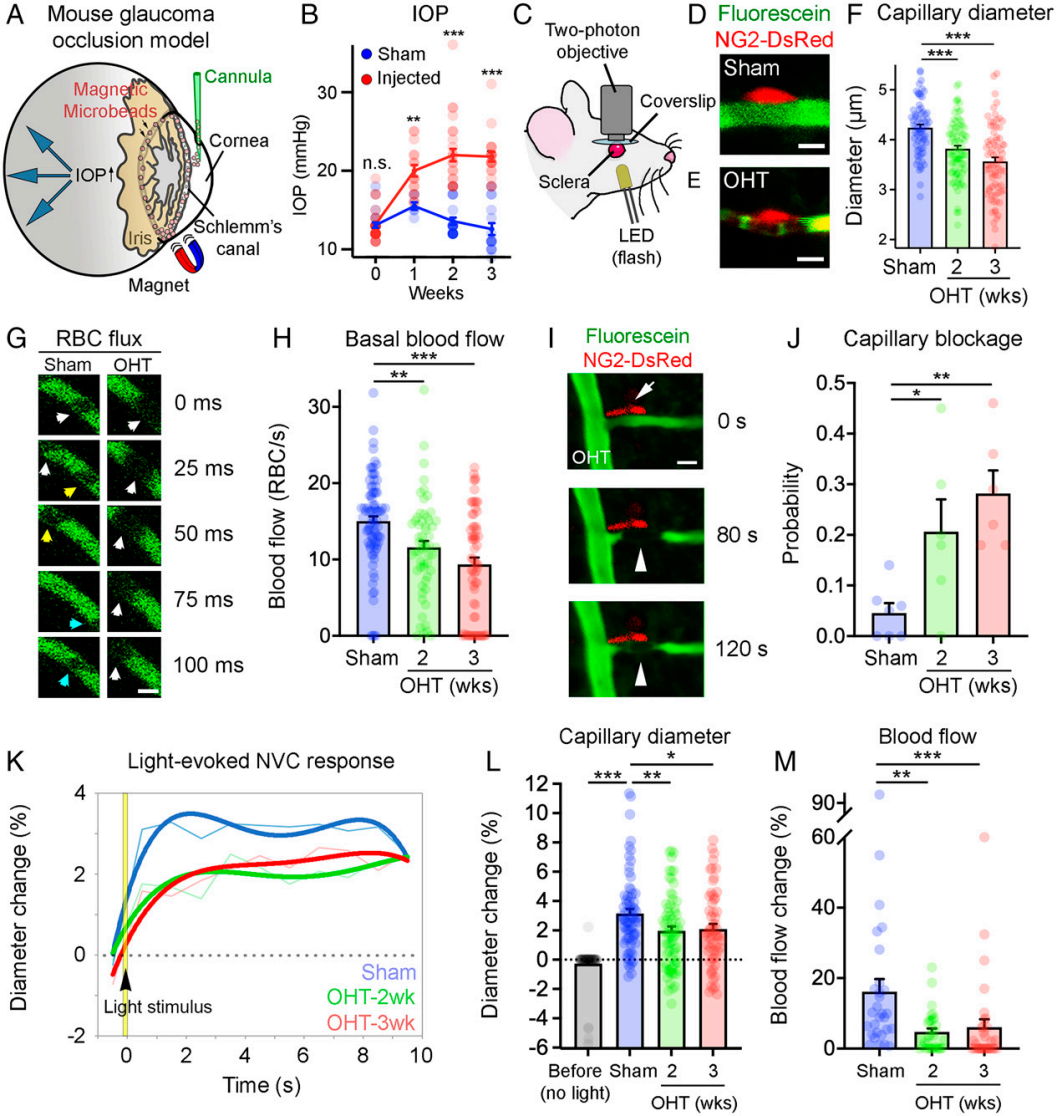
Pericyte-dependent microvascular dysfunction in glaucoma. (*A*) Schematic of magnetic microbead injection into the mouse anterior chamber to induce OHT. IOP, intraocular pressure. (*B*) This procedure results in gradual increase of intraocular pressure relative to sham-injected controls (microbead-injected: *n* = 44 mice; sham: *n* = 25 mice; two-tailed Student’s *t* test, ***P < 0.001, **P < 0.01; n.s., not significant). (*C*) Setup for TPLSM. (*D* and *E*) TPLSM live imaging of NG2-DsRed mouse retinas after injection of fluorescein (green) shows capillary constriction at pericyte locations (red) during OHT compared with sham controls. (*F*) Quantification of capillary diameter changes in vivo confirms a progressive decrease in vessel caliber at pericyte locations after OHT induction (sham: *n* = 70 capillaries, *n* = 5 mice; OHT-2 wk: *n* = 87 capillaries, *n* = 6 mice; OHT-3 wk: *n* = 77 capillaries, *n* = 6 mice; two-tailed ANOVA Tukey’s test, ***P < 0.001). (*G*) TPLSM imaging of RBC flux (RBCs per second) in single retinal capillaries. RBCs are visualized as shadows against the fluorescent background during OHT and in sham controls. Each colored arrowhead points at an individual RBC as it moves along the capillary (blood flow is shown from right to left). (*H*) Quantification of RBC flux demonstrates that basal blood flow decreases over time after OHT induction (sham: *n* = 86 capillaries, *n* = 7 mice; OHT-2 wk: *n* = 58 capillaries, *n* = 6 mice; OHT-3 wk: *n* = 64 capillaries, *n* = 6 mice; two-tailed ANOVA Tukey’s test, **P < 0.01, ***P < 0.001) (see also Movies S1, S2, and S3). (*I*) Live TPLSM imaging of an NG2-DsRed retina showing a pericyte (red, arrow) constricting a capillary (green) causing RBCs to stall, leading to capillary blockage in glaucoma (arrowheads; see also Movie S4). (*J*) The probability of capillary blockage increases significantly with OHT (sham: *n* = 86 capillaries, *n* = 7 mice; OHT-2 wk: *n* = 58 capillaries, *n* = 6 mice; OHT-3 wk: *n* = 64 capillaries, *n* = 6 mice; two-tailed ANOVA Tukey’s test, *P < 0.05, **P < 0.01). (*K*) Longitudinal analysis of light-evoked neurovascular responses in vivo shows impaired capillary vasodilation after glaucoma induction (blue: sham: *n* = 70 capillaries, *n* = 5 mice; green: OHT-2 wk: *n* = 70 capillaries, *n* = 5 mice; red: OHT-3 wk: *n* = 59 capillaries, *n* = 6 mice). NVC, neurovascular coupling. (*L*) Maximum-response graph confirms that light-triggered capillary reactivity is defective in glaucoma (before [no light] sham: *n* = 69 capillaries, *n* = 5 mice; sham: *n* = 70 capillaries, *n* = 5 mice; OHT-2 wk: *n* = 70 capillaries, *n* = 5 mice; OHT-3 wk: *n* = 59 capillaries, *n* = 6 mice; two-tailed ANOVA Dunnett’s test, *P < 0.05, **P < 0.1, ***P < 0.001). (*M*) Loss of capillary vasodilation limits blood flow availability in response to light-triggered neuronal activity (blue: sham: *n* = 29 capillaries, *n* = 5 mice; green: OHT-2 wk: *n* = 32 capillaries, *n* = 6 mice; red: OHT-3 wk: *n* = 32 capillaries, *n* = 5 mice; ANOVA on ranks Kruskal–Wallis *H* test, **P < 0.01, ***P < 0.001). Data are presented as mean values ± SEM. [Scale bars, 5 µm (*D*, *E*, and *G*) and 10 µm (*I*).]

Unilateral OHT was induced by injection of magnetic microbeads into the anterior chamber of the mouse eye. Microbeads were attracted to the iridocorneal angle with a magnet to block aqueous humor outflow and increase intraocular pressure (Fig. 1 *A* and *B*, Table 1, and *SI Appendix*, Table S1) (23). We selected two time points to characterize vascular changes in this model: 1) 2 wk after microbead injection, a time when high intraocular pressure is stable but no significant RGC loss is detected; and 2) 3 wk after the procedure, when there is measurable RGC death (18%) (*SI Appendix*, Fig. S1 *A* and *B*) (23). Capillary pericytes were visualized in mice expressing DsRed under control of the NG2 promoter using two-photon laser scanning microscopy (TPLSM), which allows minimally invasive live retinal imaging (Fig. 1*C*). Pericytes and capillaries in all vascular plexuses and branch orders were included in our analysis. High-resolution imaging of single capillaries and their associated pericytes shows a substantial reduction of capillary diameter at pericyte locations in glaucomatous eyes (OHT 2 and 3 wk) relative to sham-operated controls (3 wk) (Fig. 1 *D*–*F*). A significant capillary diameter reduction at pericytes was detected as early as 2 wk after glaucoma induction, prior to overt RGC loss. Ex vivo post hoc analysis further confirmed a substantial number of constricted capillaries at pericyte locations in glaucomatous versus sham-operated retinas (*SI Appendix*, Fig. S1 *C*–*F*). The diameter of larger vessels did not change, indicating that the observed responses were not caused by upstream arterioles (*SI Appendix*, Fig. S1*G*), and there was no reduction in retinal capillary density (*SI Appendix*, Fig. S1*H*).

**Table 1.** Intraocular pressure elevation in experimental and control groups

Time, wk	Sham-operated eyes			Microbead-injected eyes			Sham vs. injected eyes
	Mean	SEM	*N*	Mean	SEM	*N*	*P* value(two-tailed Student’s *t* test)
0	13.1	0.3	25	13.2	0.3	44	0.3526
1	15.5	0.5	6	20.0	0.8	16	0.0034
2	13.6	0.5	25	22.0	0.8	20	<0.0001
3	12.6	0.8	16	21.8	0.6	20	<0.0001

Next, we asked whether reduction of capillary diameter at pericyte locations affected the microcirculation in glaucoma. Single-capillary blood flow was measured using TPLSM by quantification of the number of red blood cells (RBCs) per second (15, 24) (Fig. 1*G* and Movie S1). We observed a significant reduction of capillary blood flow in glaucomatous eyes compared with sham controls (Fig. 1*H* and Movies S1, S2, and S3). Live TPLSM visualization showed that reduced blood flow correlated with pericyte-induced capillary constriction (Fig. 1*I* and Movie S4). Furthermore, the probability of capillary blood flow interruption at pericyte locations increased by fivefold in glaucoma relative to sham controls (Fig. 1*J*). We then examined whether retinal neurovascular coupling was affected by high intraocular pressure. For this purpose, we used TPLSM to record capillary dynamics and blood flow before and after light stimulation in vivo. The ability of capillaries to dilate in response to light was severely compromised in glaucoma relative to sham-operated controls (Fig. 1*K*). Consistent with impaired neurovascular coupling, we observed a marked reduction in light-evoked dilation and blood flow in glaucomatous eyes (Fig. 1 *L* and *M*). Light-evoked dilation was not due to artifactual movements in the *z* axis since volume imaging of capillaries during light stimulation showed similar results as single-plane measurements (*SI Appendix*, Fig. S1*I*). No vascular deficits were observed in sham-operated controls using identical imaging conditions (Fig. 1 *F*, *H*, and *J*–*M*), thus ruling out artifacts or adverse effects of the TPLSM approach used here. Together, these results indicate that pericytes play a crucial role in microvascular pathology in glaucoma, notably decreased capillary diameter, reduced blood flow, and impaired neurovascular coupling.

### Interpericyte Communication Is Compromised by High Intraocular Pressure.

**Fig. 2.**
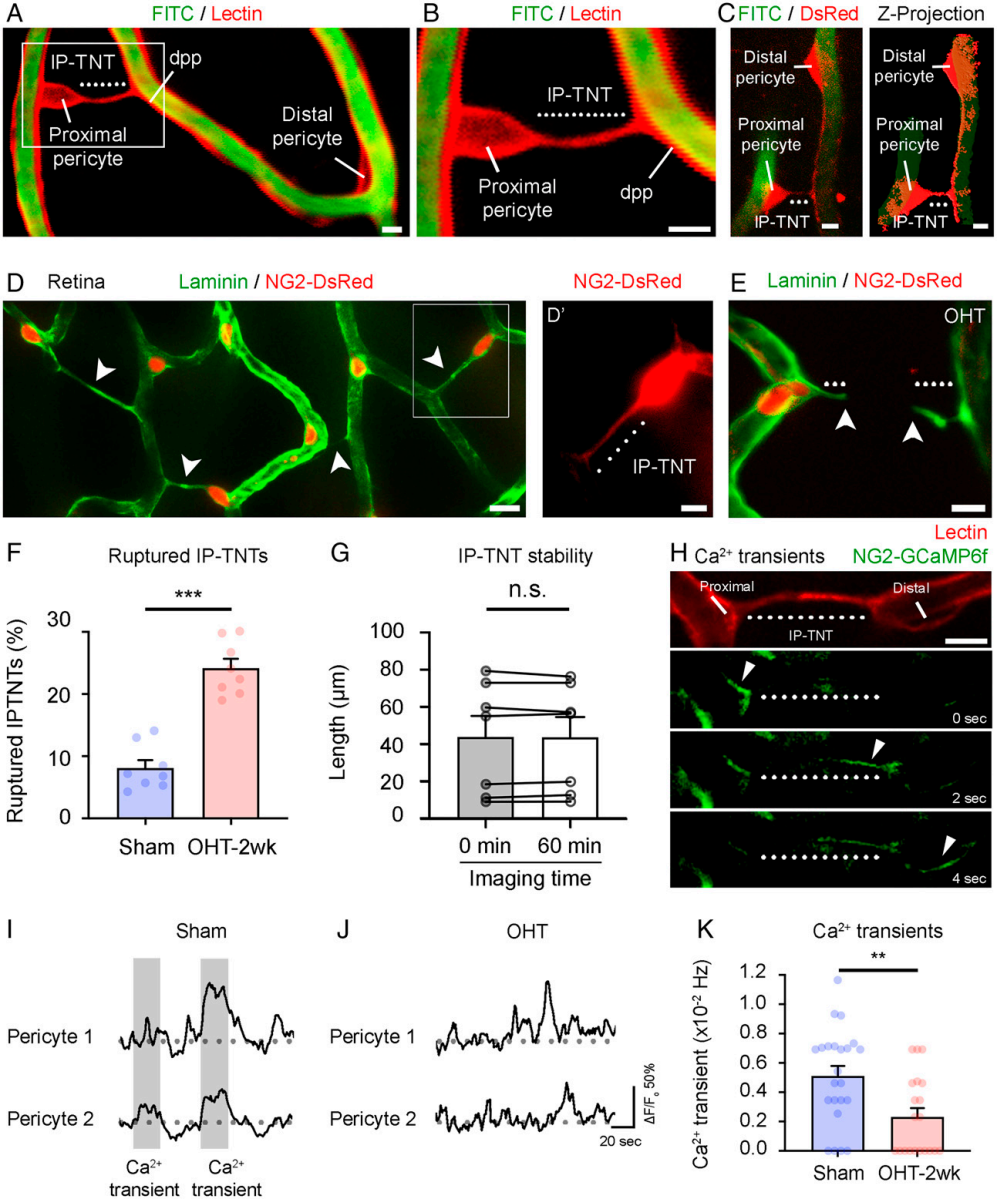
Interpericyte communication is compromised by high intraocular pressure. (*A*) IP-TNT (dotted line) extending from a pericyte soma, visualized with TRITC-tagged lectin, connects to a distal pericyte process (dpp) located on a neighboring capillary, visualized after tail-vein injection of FITC-coupled dextran. (*B*) Higher magnification of the *Inset* in *A*. (*C*) Example of an IP-TNT and its *z* projection (*Right*), this time visualized in an NG2-DsRed retina, linking a proximal and a distal pericyte. (*D* and *D′*) Retinal immunostaining with laminin shows a network of IP-TNTs linking distal capillaries. Endogenous DsRed can be seen at higher magnification (*Inset* and *D′*). Arrowheads indicate IP-TNTs within a capillary network (*D*). (*E* and *F*) OHT leads to retinal IP-TNT rupture (sham: *n* = 1,356 IP-TNTs, *n* = 8 mice; OHT-2 wk: *n* = 918 IP-TNTs, *n* = 8 mice; two-tailed Student’s *t* test, ***P < 0.001). Arrowheads mark rupture points of IP-TNT (*E*). (*G*) IP-TNTs were stable in noninjured controls and did not change over time despite imaging periods of up to 60 min (*n* = 7 IP-TNTs, *n* = 6 mice; two-tailed paired Student’s *t* test; n.s., not significant). (*H*) Time-lapse recordings of Ca^2+^ transients in an IP-TNT labeled with TRITC-tagged lectin and its associated pericytes, visualized in an NG2-GCaMP6f mouse (see also Movies S5, S6, and S7). Arrowheads follow the movement of a Ca2+ transient between pericytes in time. (*I*) Ca^2+^ transients are measured as synchronous Ca^2+^ increases in IP-TNT–coupled pericyte pairs (gray shading). (*J* and *K*) The frequency of interpericyte Ca^2+^ transients is reduced during OHT compared with sham-operated control eyes (sham: *n* = 23 IP-TNTs, *n* = 6 mice; OHT-2 wk: *n* = 20 IP-TNTs, *n* = 5 mice; two-tailed Student’s *t* test, **P < 0.01). Data are presented as mean values ± SEM. [Scale bars, 5 µm (*A–C*, *D′*, *E*, and *H*) and 10 µm (*D*).]

To elucidate mechanisms underlying vascular alterations in glaucoma, we focused primarily on changes at 2 wk after glaucoma induction, which preceded significant neuronal death (*SI Appendix*, Fig. S1 *A* and *B*), thus better reflecting early pathology onset. We recently identified IP-TNTs, nanotube-like processes mediating pericyte-to-pericyte communication in the retina, which control local neurovascular coupling and coordinate light-evoked responses between adjacent capillaries (15). IP-TNTs are abundant in all vascular plexuses of the retina (deep, intermediate, superficial) (15). To test whether neurovascular coupling impairment in glaucoma involved IP-TNTs, we first examined IP-TNT structural changes in all vascular plexuses of hypertensive and control eyes using live imaging. In noninjured control retinas, colocalization of pericyte-specific DsRed or tetramethylrhodamine-5-(and 6-)isothiocyanate (TRITC)–lectin with fluorescein isothiocyanate (FITC)–coupled dextran (to label capillaries) showed IP-TNTs emerging from the pericyte soma and connecting with a distal pericyte process (Fig. 2 *A*–*C*). In healthy sham retinas, IP-TNTs formed networks linking pericytes on separate capillary systems (Fig. 2 *D* and *D′*). In contrast, we observed a substantial number of damaged or ruptured IP-TNTs in glaucomatous retinas, accounting for 25% of all IP-TNTs (Fig. 2 *E* and *F*). IP-TNTs were stable in noninjured controls and did not change over time despite imaging periods of up to 60 min (the longest time tested) (Fig. 2*G*), ruling out any changes due to the TPLSM technique itself. Intraocular pressure–dependent force at the optic nerve head has been proposed to exert biomechanical stress on the lamina cribrosa, which can damage RGC axons (25). Due to its location deeper behind the orbit, it was not possible to image the optic nerve head by TPLSM, but ex vivo analysis of this region confirmed the presence of IP-TNTs linking pericytes across capillaries (*SI Appendix*, Fig. S2 *A* and *A′*). Similar to the retina, IP-TNTs within the optic nerve head were damaged in glaucoma (*SI Appendix*, Fig. S2 *B* and *C*). IP-TNTs mediate pericyte-to-pericyte communication through (Ca^2+^) transients that are essential for the coordination of capillary responses (15). Ca^2+^ transients were measured as spontaneous Ca^2+^ increases in IP-TNT–coupled pericytes imaged in mice expressing the Ca^2+^ indicator GCaMP6f downstream of the NG2 promoter (NG2-GCaMP6f) both in live retinas by TPLSM and retinal explants (Fig. 2 *H* and *I* and Movies S5, S6, and S7). A marked decrease in the frequency of these Ca^2+^ transients was observed following induction of OHT (Fig. 2 *J* and *K*), consistent with IP-TNT damage. Taken together, our results indicate that the structural and functional integrity of IP-TNTs, including their ability to serve as a communication conduit between linked pericytes, is compromised in glaucoma.

### Excessive Ca^2+^ Influx to Pericytes Mediates Neurovascular Deficits.

**Fig. 3.**
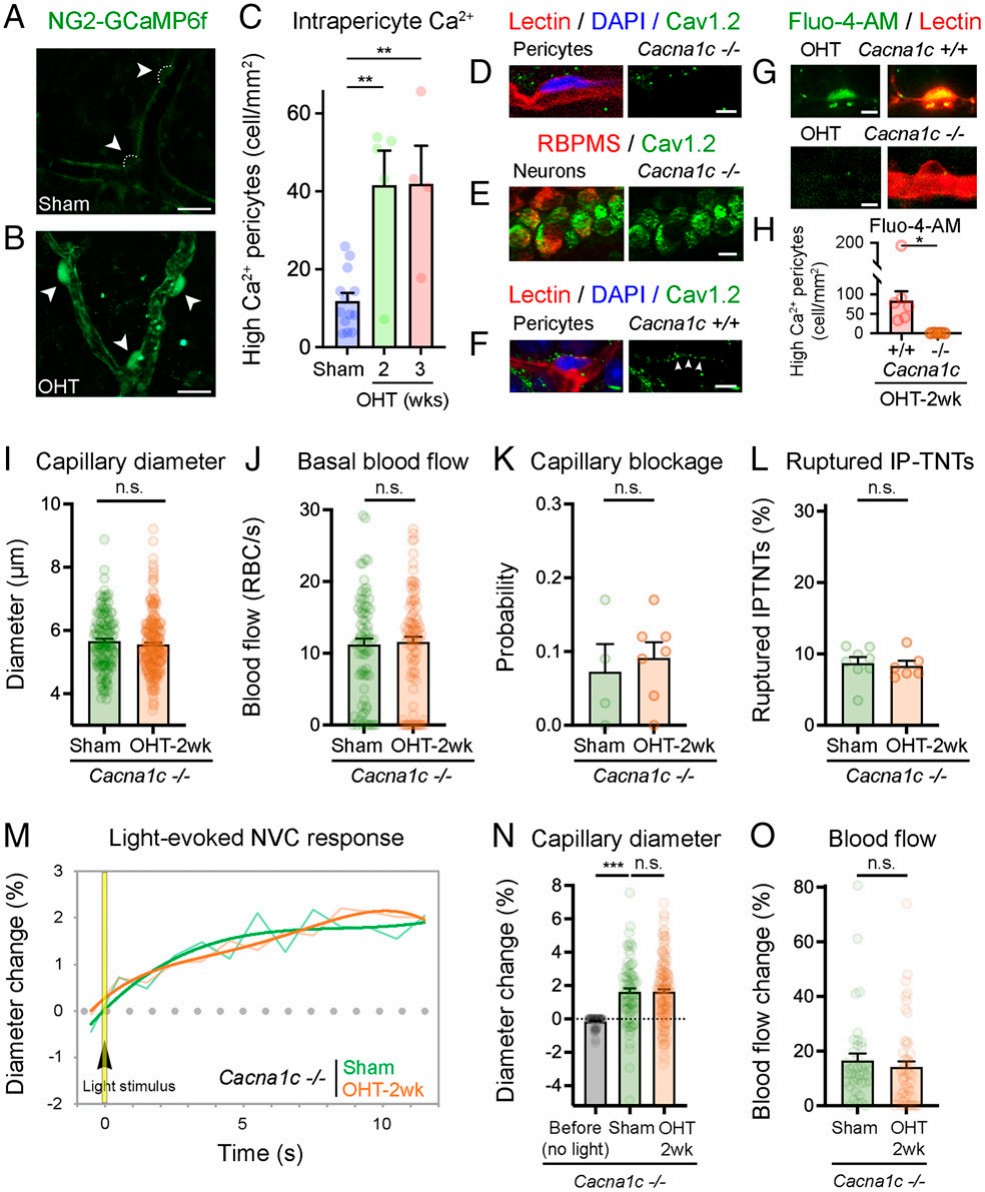
Excessive Ca^2+^ influx to pericytes mediates neurovascular deficits. (*A* and *B*) Analysis of NG2-GCaMP6f retinas demonstrates a substantial increase of Ca^2+^ in pericytes from eyes subjected to glaucoma relative to sham-operated controls (arrowheads). (*C*) Quantitative analysis shows a marked increase in intrapericyte Ca^2+^ signals with OHT relative to control eyes (sham: *n* = 235 pericytes, *n* = 13 mice; OHT-2 wk: *n* = 168 pericytes, *n* = 5 mice; OHT-3 wk: *n* = 182 pericytes, *n* = 4 mice; two-tailed ANOVA Tukey’s test, **P < 0.01). (*D* and *E*) Immunohistochemistry confirms the absence of Cav1.2 protein in pericytes from *Cacna1c^−/−^* retinas, while adjacent RGCs visualized with the cell-specific marker RBPMS contain Cav1.2 serving as an internal positive control. (*F*) Wild-type littermate control mice (*Cacna1c*^+/+^) show abundant Cav1.2 expression in pericytes. Arrowheads indicate Cav1.2 expression (green) in pericytes. (*G* and *H*) Pericytes in wild-type littermate control retinas (*Cacna1c*^+/+^) display a marked increase of intracellular Ca^2+^ after glaucoma induction, visualized with the Ca^2+^ indicator Fluo-4-AM, whereas pericytes in *Cacna1c*^−/−^ retinas show only basal Ca^2+^ levels, supporting Cav1.2 depletion (*Cacna1c*^+/+^: *n* = 6 mice; *Cacna1c*^−/−^: *n* = 5 mice; two-tailed Student’s *t* test, *P < 0.05). (*I* and *J*) Selective deletion of *Cacna1c* in pericytes resulted in the preservation of capillary diameter (*I*; sham: *n* = 135 capillaries, *n* = 5 mice; OHT-2 wk: *n* = 223 capillaries, *n* = 8 mice; two-tailed Student’s *t* test; n.s., not significant) and blood flow (*J*; sham: *n* = 75 capillaries, *n* = 4 mice; OHT-2 wk: *n* = 95 capillaries, *n* = 7 mice; Mann–Whitney *U* test; n.s., not significant). (*K* and *L*) The probability of capillary blockage was substantially reduced in *Cacna1c*^−/−^ retinas (*K*; sham: *n* = 75 capillaries, *n* = 4 mice; OHT-2 wk: *n* = 95 capillaries, *n* = 7 mice; two-tailed Student’s *t* test; n.s., not significant), and IP-TNTs were protected in the absence of *Cacna1c* (*L*; sham: *n* = 1,563 IP-TNTs, *n* = 8 mice; OHT-2 wk: *n* = 1,071 IP-TNTs, *n* = 6 mice; two-tailed Student’s *t* test; n.s., not significant). (*M–O*) Light-evoked neurovascular responses were maintained in glaucomatous *Cacna1c*^−/−^ mice relative to sham controls including capillary hemodynamic and maximum responses (*M* and *N*; before [no light] sham: *n* = 81 capillaries, *n* = 5 mice; sham: *n* = 81 capillaries, *n* = 5 mice; OHT-2 wk: *n* = 152 capillaries, *n* = 7 mice; two-tailed ANOVA Tukey’s test, ***P < 0.001; n.s., not significant) as well as blood flow (*O*; sham: *n* = 39 capillaries, *n* = 4 mice; OHT-2 wk: *n* = 55 capillaries, *n* = 7 mice; Mann–Whitney *U* test; n.s., not significant). Data are presented as mean values ± SEM. [Scale bars, 10 µm (*A*, *B*, and *E*) and 5 µm (*D*, *F*, and *G*).]

In addition to playing a key role in pericyte-to-pericyte communication, cytosolic Ca^2+^ regulates the contractile activity of pericytes (26). To investigate whether alterations in intrapericyte Ca^2+^ promoted neurovascular dysfunction during glaucomatous stress, we examined Ca^2+^ signals in NG2-GCaMP6f mice both in vivo by TPLSM imaging and ex vivo in retinal explants (Fig. 3 *A*–*C*). OHT induced a robust and sustained increase in global Ca^2+^ within pericytes, while little cytosolic intrapericyte Ca^2+^ was detected in sham-operated controls (Fig. 3 *A*–*C*). A major path of Ca^2+^ influx in pericytes is through L-type voltage-gated Ca^2+^ channels (27), of which the alpha 1C subunit (Cav1.2) is enriched in pericytes (28). To test whether excessive intrapericyte Ca^2+^ played a role in microvascular deficits, we generated mice with a pericyte-specific conditional deletion of the gene encoding Cav1.2 (*Cacna1c*). Immunohistochemical analysis of retinas from *Cacna1c*-null mice (*Cacna1c^−/−^*) confirmed the selective absence of Cav1.2 in pericytes, whereas adjacent neurons were positive for Cav1.2, thus serving as internal controls (Fig. 3 *D* and *E*). Wild-type littermate control mice (*Cacna1c^+/+^*) expressed Cav1.2 in both pericytes and neurons (Fig. 3*F*). Analysis of Ca^2+^ levels using Fluo-4-AM confirmed lack of glaucoma-induced Ca^2+^ increase in pericytes from *Cacna1c^−/−^* retinas, which displayed basal Ca^2+^ levels similar to sham controls, whereas substantial intrapericyte Ca^2+^ levels were detected in *Cacna1c^+/+^* retinas (Fig. 3 *G* and *H*). Fluo-4-AM allowed reliable detection of Ca^2+^ levels in pericytes (*Materials and Methods*), but lacked sensitivity to enable the measurement of Ca^2+^ dynamics in pericytes and IP-TNTs. To rule out abnormalities caused by *Cacna1c* deletion, we examined the structure of retinal layers and RGCs as well as vascular parameters in noninjured *Cacna1c^−/−^* and *Cacna1c^+/+^* mice. Our data demonstrate that Cav1.2 depletion did not lead to major defects in the thickness of retinal layers, RGC density, or soma size, ruling out developmental defects (*SI Appendix*, Figs. S3 *A*–*H* and S5*C*). We found a slight increase in basal capillary diameter in noninjured *Cacna1c^−/−^* retinas relative to *Cacna1c^+/+^* controls, which likely reflects the effect of reduced pericyte contractility on capillary tone, but basal blood flow, capillary blockade probability, and total number of IP-TNTs were similar, thus confirming the absence of major vascular alterations (*SI Appendix*, Fig. S3 *I*–*L*).

Next, we examined capillary and blood flow changes in *Cacna1c^−/−^* mice and wild-type littermate controls following induction of OHT or sham surgery. Conditional pericyte-specific *Cacna1c* deletion did not alter microbead-induced pressure elevation (*Cacna1c^−/−^*: 21 ± 1 mm Hg, *n* = 6 mice; *Cacna1c^+/+^*: 23 ± 2 mm Hg, *n* = 5 mice; Student’s *t* test, *P* = 0.3289). Our data demonstrate that restoration of Ca^2+^ homeostasis in pericytes lacking *Cacna1c* preserved capillary diameter and blood flow in glaucoma, in addition to reducing the likelihood of capillary blockage, to levels found in sham controls (Fig. 3 *I*–*K*). We also found that IP-TNTs were protected in glaucomatous *Cacna1c^−/−^* retinas and optic nerves (Fig. 3*L* and *SI Appendix*, Fig. S2 *D* and *E*), and that light-evoked hemodynamic responses in *Cacna1c^−/−^* mice with OHT were similar to sham controls (Fig. 3 *M*–*O*). In contrast, wild-type littermate mice subjected to glaucoma underwent significant capillary diameter and blood flow reduction, increased capillary blockage, IP-TNT damage, and loss of neurovascular responses (*SI Appendix*, Fig. S4). Collectively, these results demonstrate that 1) excessive intrapericyte Ca^2+^ promotes vascular dysregulation, and 2) pericyte-specific Cav1.2 deletion restores intrapericyte Ca^2+^ homeostasis, capillary dynamics, blood flow, and neurovascular coupling in glaucoma.

### Recovery of Ca^2+^ Homeostasis in Pericytes Restores Light-Evoked Neuronal Responses and Promotes RGC Survival.

**Fig. 4.**
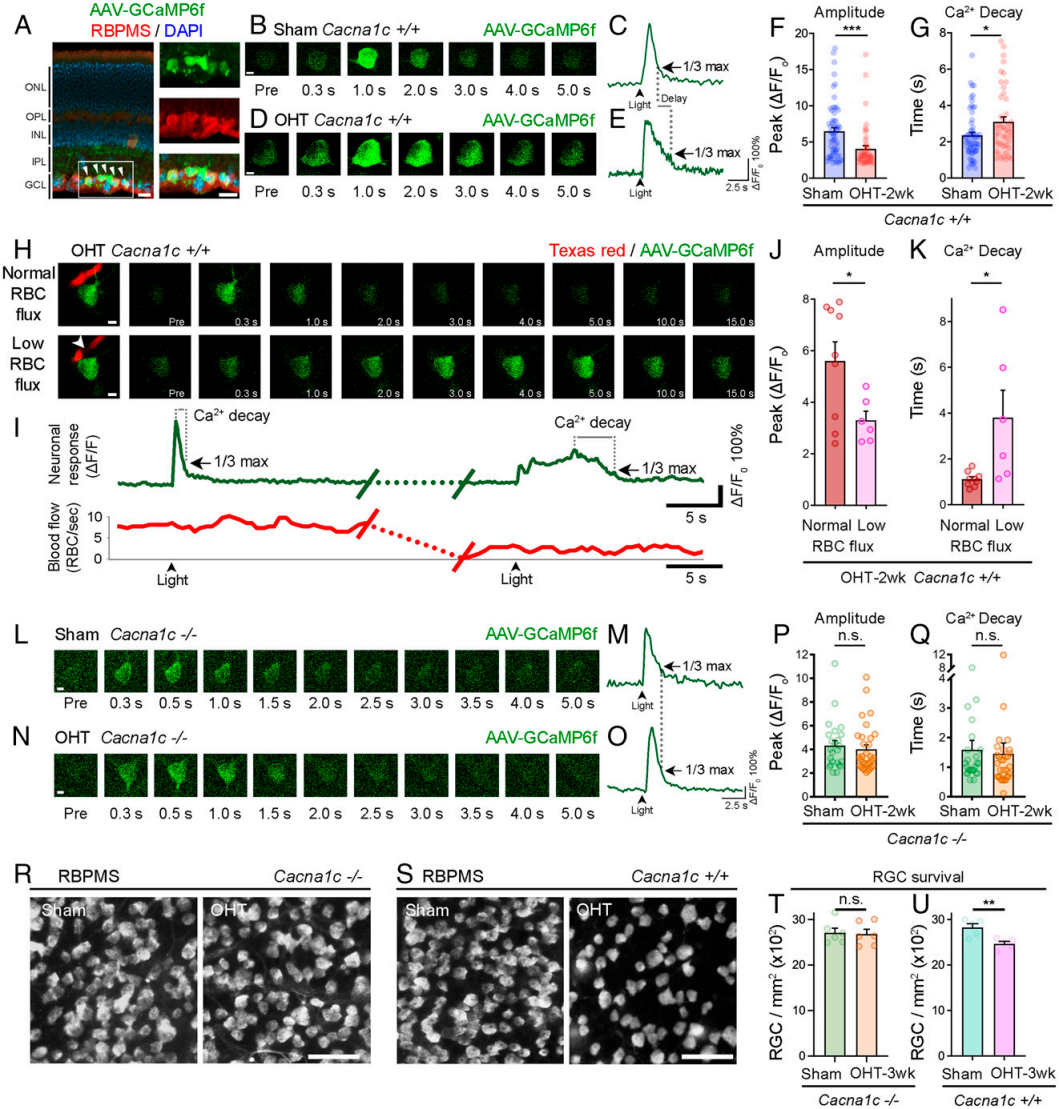
Recovery of Ca^2+^ homeostasis in pericytes restores light-evoked neuronal responses and promotes RGC survival. (*A*) Retinal cross-section shows AAV-mediated expression of GCaMP6f (green) in RGCs, confirmed by colocalization with the RGC-specific marker RBPMS (red). Nuclei are labeled with DAPI (blue). Arrowheads in indicate AAV-mediated GCaMP6f (green) expression in RGC. (*B* and *C*) Light stimulation of a GCaMP6f-positive αON-S RGC elicits a brief burst of activity measured as an increase in Ca^2+^ signal followed by rapid recovery in wild-type control retinas (*Cacna1c^+/+^*). (*D–G*) A marked reduction of RGC Ca^2+^ signal amplitude and delayed recovery (Ca^2+^ decay) is observed in ocular hypertensive *Cacna1c^+/+^* eyes (*F*; amplitude; sham: *n* = 61 RGCs, *n* = 12 mice; OHT: *n* = 48 RGCs, *n* = 4 mice; Mann–Whitney *U* test, ***P < 0.001) (*G*; Ca^2+^ decay; sham: *n* = 61 RGCs, *n* = 12 mice; OHT: *n* = 45 RGCs, *n* = 4 mice; Mann–Whitney *U* test, *P < 0.05). (*H* and *I*) Simultaneous time-lapse TPLSM recordings and representative traces of light-evoked Ca^2+^ responses of a single RGC transduced with AAV-GCaMP6f (green trace), and blood flow (red trace) of the main capillary feeding the same neuron (intravenous Texas red; red trace) before and after OHT-induced vessel constriction. The arrowhead shows a trapped RBC (see also Movie S8). The data shown in *H* and *I* correspond to the same vessel and neuron. (*J* and *K*) When capillary blood flow is within the normal range, neuronal Ca^2+^ responses are robust and decay rapidly but, when blood flow is compromised, peak amplitude response is reduced and decay time increases (*J*; peak amplitude; normal RBC flux: *n* = 9 neurons/capillaries, *n* = 5 mice; low RBC flux: *n* = 6 neurons/capillaries, *n* = 5 mice; two-tailed Student’s *t* test, *P < 0.05) (*K*; Ca^2+^ decay time; normal RBC flux: *n* = 9 neurons/capillaries, *n* = 5 mice; low RBC flux: *n* = 6 neurons/capillaries, *n* = 5 mice; two-tailed Student’s *t* test, *P < 0.05). (*L–Q*) Light-evoked RGC Ca^2+^ responses are protected in glaucomatous *Cacna1c*-null mice displaying parameters similar to those in sham-operated controls (*P*; peak amplitude; sham: *n* = 24 RGCs, *n* = 6 mice; OHT-2 wk: *n* = 31 RGCs, *n* = 6 mice; Mann–Whitney *U* test; n.s., not significant) (*Q*; Ca^2+^ decay time; sham: *n* = 24 RGCs, *n* = 6 mice; OHT-2 wk: *n* = 31 RGCs, *n* = 6 mice; Mann–Whitney *U* test; n.s., not significant). (*R*–*U*) Cav1.2 deletion promotes RGC survival and preserves neuronal density at levels similar to noninjured sham-operated control eyes, whereas substantial RGC loss occurs in wild-type littermate controls (*T*; sham: *n* = 6 mice; OHT-3 wk: *n* = 6 mice; two-tailed Student’s *t* test; n.s., not significant) (*U*; sham: *n* = 5 mice; OHT-3 wk: *n* = 5 mice; two-tailed Student’s *t* test, **P < 0.01). Data are presented as mean values ± SEM. GCL, ganglion cell layer; INL, inner nuclear layer; IPL, inner plexiform layer; ONL, outer nuclear layer; OPL, outer plexiform layer. [Scale bars, 20 µm (*A*), 5 µm (*B*, *D*, *H*, *L*, and *N*), and 50 µm (*R* and *S*).]

To evaluate whether improved pericyte and vascular function in *Cacna1c^−/−^* mice affected RGC activity, we recorded light-evoked single-RGC Ca^2+^ responses by TPLSM using an adeno-associated virus (AAV) encoding GCaMP6f driven by the synapsin promoter (AAV-GCaMP6f). Selective GCaMP6f expression in RGC was confirmed on retinal flat mounts and cross-sections using the RGC-specific marker RBPMS (RNA-binding protein with multiple splicing) (29) (Fig. 4*A* and *SI Appendix*, Fig. S5*A*). We focused on alpha ON-sustained (αON-S) RGCs, a major cell class characterized by maintained firing during the bright phase (*SI Appendix*, Fig. S5*B*) (30, 31). The identity of αON-S RGCs was further confirmed by post hoc analysis of soma area, dendritic stratification in the proximal ON sublamina b, and high levels of neurofilament H protein (SMI-32) (*SI Appendix*, Fig. S5 *C*–*G*) (30–32). To correlate vascular changes with RGC function, Ca^2+^ responses were assessed using the same stimulation protocol employed in neurovascular coupling tests (single flash, 6 ms). In wild-type control retinas, GCaMP6f-positive αON-S RGCs elicited a brief burst of light-evoked Ca^2+^ signal followed by a rapid signal decay or recovery (Fig. 4 *B* and *C*). In contrast, a marked reduction of Ca^2+^ signal amplitude and delayed recovery, defined as the time to fall to one-third of the Δ*F/F* peak maximum response, was observed in ocular hypertensive eyes (Fig. 4 *D*–*G*). To establish whether compromised blood supply in glaucoma affected neuronal function, we simultaneously imaged by TPLSM light-evoked Ca^2+^ responses in αON-S RGCs and blood flow in the adjacent capillaries in wild-type mice. Using this approach, we longitudinally recorded single-RGC Ca^2+^ responses with blood flow in the capillary serving the same neuron before and after pericyte-induced vessel constriction during glaucoma. Fig. 4 *H* and *I* show representative recordings and traces obtained from the same vessel and neuron. Our data demonstrate that when retinal capillary blood flow was within the normal range (7 to 20 RBCs per second), consistent with capillary blood flow measurements in the brain (24), RGC Ca^2+^ responses were robust and decayed rapidly. However, when blood flow was compromised (0 to 7 RBCs per second), Ca^2+^ signals were reduced and recovery was significantly delayed (Fig. 4 *H* and *I* and Movie S8). Quantitative analysis of Ca^2+^ signal parameters confirmed a significant reduction in the peak amplitude response and increased decay time in neurons fed by capillaries with reduced blood flow (Fig. 4 *J* and *K*). These changes were not due to altered Ca^2+^ dynamics or refractoriness caused by recurrent light stimuli because the same stimulation protocol did not alter RGC responses in wild-type sham controls with normal blood flow (*SI Appendix*, Fig. S5 *H*–*J*). Next, we investigated whether preventing vascular abnormalities rescued RGC function in ocular hypertensive eyes by measuring light-evoked Ca^2+^ responses in RGCs from *Cacna1c^−/−^* mice. Our results demonstrate that, contrary to wild-type mice showing significant alterations in αON-S RGC Ca^2+^ dynamics after glaucoma induction (i.e., reduced amplitude, delayed recovery) (Fig. 4 *D*–*G*), *Cacna1c^−/−^* mice were protected and displayed healthy Ca^2+^ responses (Fig. 4 *L*–*Q*). Lastly, we examined whether recovery of pericyte function after pericyte-specific deletion of Cav1.2 had an impact on neuronal survival. RGC soma density in *Cacna1c^−/−^* and *Cacna1c^+/+^* retinas was quantified at 3 wk of OHT, a time when there is significant RGC loss, thus allowing the assessment of neuroprotection (*SI Appendix*, Fig. S1 *A* and *B*). Cav1.2 deletion promoted RGC survival and, strikingly, preserved neuronal density at a level similar to that found in noninjured sham-operated control eyes. In contrast, substantial RGC death was observed in wild-type littermate controls (Fig. 4 *R*–*U*). We conclude that reducing Ca^2+^ influx in pericytes restores RGC function and promotes cell survival, suggesting that capillary dysfunction directly impairs neuronal activity and compromises RGC viability in glaucoma.

## Discussion

Glaucoma is a complex and multifactorial neurodegenerative disease. There are two main forms of the adult-onset pathology based on the mechanism of aqueous humor outflow obstruction: open-angle glaucoma (OAG) and angle-closure glaucoma. OAG, the most common type, is characterized by slow and asymptomatic damage to the optic nerve that correlates with elevated intraocular pressure (33). OAG includes a subgroup of patients who develop optic neuropathy in conditions of normal or low intraocular pressure, known as normal-tension glaucoma (34). In OAG, increased resistance to aqueous humor outflow at the trabecular meshwork leads to OHT, but the structures in the anterior chamber angle are visible by gonioscopy (i.e., trabecular meshwork, scleral spur, ciliary body band) (33). In contrast, angle-closure glaucoma can develop suddenly from an acute increase in intraocular pressure when the iris, abnormally positioned, blocks the outflow pathway. The OHT model used in our study recapitulates the features of conventional OAG because 1) it involves a gradual, rather than acute, increase in intraocular pressure above normal levels; 2) the deposition of magnetic microbeads at the trabecular meshwork effectively increases the resistance to aqueous humor outflow; and 3) other iridocorneal angle structures are unaltered. Many glaucoma patients suffer from vascular abnormalities thought to contribute to visual field loss, but the molecular basis of these deficits and how they impact RGC function are poorly understood. In this study, we identify pericytes as key mediators of capillary dysfunction in eyes with high intraocular pressure. Our data show that pericytes constrict retinal capillaries, substantially reducing blood flow, and that IP-TNT damage impairs neurovascular coupling in glaucomatous eyes. Blockade of Ca^2+^ influx to pericytes preserved capillary dynamics, improved blood flow, protected IP-TNTs, and rescued neurovascular coupling during ocular hypertensive stress. Importantly, the restoration of microvascular function by selectively reducing intrapericyte Ca^2+^ restored light-evoked RGC responses and promoted neuronal survival. Together, these findings reveal that pathological changes in pericytes caused by loss of Ca^2+^ homeostasis play a critical role in microcirculatory blood flow deficits that contribute to RGC dysfunction and damage in glaucoma.

Until recently, cerebral blood flow was believed to be solely regulated by smooth muscle cells on arterioles. In line with this, most studies on vascular dysregulation in glaucoma patients have focused on changes at the level of arterioles (6, 35). Recent studies, however, demonstrate not only that capillaries offer the highest resistance to blood supply but that capillary pericytes express contractile proteins and hence contract and relax to control blood microcirculation (16, 18, 19, 36–38). From this perspective, it is essential to understand the role of pericytes in diseases with a vascular component such as glaucoma. Here, we capitalized on our ability to use TPLSM live imaging together with molecular tools to monitor real-time capillary changes at pericyte locations during glaucomatous damage. This approach allowed high spatiotemporal resolution of basal as well as light-evoked capillary dynamics and blood flow. We report substantial pericyte-induced capillary constriction and reduced or stalled blood flow in glaucoma, a response that did not involve changes in upstream arterioles (*SI Appendix*, Fig. S1*G* and Movie S4). Optogenetic activation of brain capillary pericytes was recently shown to constrict capillaries and reduce blood flow (16, 17). Pericyte-mediated capillary constriction was also reported after transient retinal ischemia, a condition in which capillary narrowing persisted after reperfusion (15, 22). Capillary constriction by pericytes has also been observed in brain biopsy tissue from Alzheimer’s disease (AD) patients with Aβ deposition and in a mouse model of AD (39). Together, these findings support a critical role of pericytes in the physiological regulation of capillary blood flow in both the retina and brain as well as in the context of neuropathologies.

Information processing in the visual pathway depends critically on neurovascular coupling, the ability to deliver adequate blood supply to meet the energy requirement of neurons. Multiple mechanisms regulate neurovascular coupling, notably enzyme-derived mediators such as nitric oxide and arachidonic acid derivatives as well as adenosine triphosphate and K^+^ acting on astrocytes and endothelial cells, respectively (24, 40). However, until recently, the mechanism by which retinal capillaries synchronize and distribute blood during neurovascular coupling was unknown. In this regard, we demonstrated that retinal IP-TNTs coordinate blood flow changes between linked capillaries and that selective IP-TNT ablation eliminates coupled neurovascular responses (15). The discovery of IP-TNTs underscores the spatial and temporal heterogeneity of blood redistribution within retinal capillary networks, where a limited amount of blood is rapidly relocated to meet the demand of active versus inactive neurons (41). Here, we found that many IP-TNTs are damaged or dysfunctional in glaucoma, as evidenced by the loss of pericyte-to-pericyte communication and impaired light-evoked neurovascular coupling. We previously demonstrated that the frequency of Ca^2+^ transients between pericytes was substantially reduced after IP-TNT ablation and administration of gap-junction blockers, consistent with our observation that IP-TNTs connect with distal pericyte processes via gap junctions (15). Further work is needed to establish whether interpericyte communication occurs via intercellular Ca^2+^ waves (42, 43) or other mechanisms. Collectively, our findings are consistent with studies showing that patients with primary OAG have impaired vascular reactivity, notably reduced light-evoked vasodilation and sluggish neurovascular responses (9, 11, 12, 44–47). The conventional view in the glaucoma field is that vascular deficits are secondary to neuronal loss (6, 48, 49). We show here that vascular pathology occurs at early stages of the disease, hence raising a critical question: Can pericyte/capillary defects directly cause neuronal dysfunction? To test this, we recorded Ca^2+^ dynamics in single αON-S RGCs by TPLSM and found that these responses were altered in glaucoma. In a key experiment, we longitudinally followed the same neuron and its serving capillary before and after blood flow interruption. Our data demonstrate that when blood flow was normal, αON-S displayed a predictable light-evoked Ca^2+^ response; however, as soon as blood supply was compromised, Ca^2+^ dynamics were severely altered. In particular, the observed increase in the Ca^2+^ decay time in glaucoma, which can result in sustained cytosolic Ca^2+^ accumulation, can subsequently contribute to RGC death via dysregulation of survival signaling pathways and activation of apoptosis (50–52). Together, these findings raise the intriguing possibility that pericyte-induced blood flow reduction, which hinders oxygen and nutrient supply to energetically demanding RGCs (53), triggers neuronal dysfunction, thus sensitizing neurons to pressure-related stressors. Given that glaucoma is a chronic disease spanning several decades of a patient’s life, our data suggest that sustained pericyte-induced neurovascular impairment will negatively impact RGC function and long-term viability.

A critical property of pericytes is their ability to contract and relax to regulate capillary blood flow (19), a process enabled by the presence of contractile proteins such as α-SMA (18). Pericytes are electrically excitable cells finely tuned by Ca^2+^-permeable membrane channels and intracellular Ca^2+^ stores (26). Ca^2+^ influx induces conformational changes in the myosin–actin complex, leading to pericyte contraction, a response observed in many systems including the retina (54). We show that intrapericyte Ca^2+^ levels substantially increase in glaucomatous retinas. The L-type voltage-dependent Ca^2+^ channel is an important mediator of Ca^2+^ influx in pericytes (26) and single-cell RNA sequencing revealed that this channel’s Cav1.2 subunit is enriched in pericytes (28). Our data demonstrate that conditional deletion of the gene encoding Cav1.2 in pericytes preserved capillary diameter and blood flow in ocular hypertensive eyes. Furthermore, in the absence of Cav1.2, IP-TNTs were protected and light-evoked hemodynamic responses were restored in glaucomatous retinas. These findings indicate that excessive Ca^2+^ influx to pericytes via L-type voltage-dependent Ca^2+^ channels plays a major role in the pathogenesis of capillary defects in glaucoma. Whether pericyte-specific Cav1.2-containing Ca^2+^ channels are preferentially active or up-regulated in glaucoma remains to be determined. Importantly, we show that when pathological Ca^2+^ influx to pericytes is restricted, RGC function is restored, leading to enhanced survival, thus identifying pericytes as promising therapeutic targets for glaucoma and potentially other optic neuropathies.

There are several limitations to our study. First, the effect of pericyte-mediated capillary defects on neuronal activity focused on αON-S RGCs, a major neuronal class. Nonetheless, it is possible that other RGC subtypes respond differently to microcirculatory deficits. Indeed, various degrees of RGC susceptibility to optic nerve injury have been reported (32, 55). Therefore, it will be of interest to characterize the response of other RGC subtypes to vascular damage. Second, we focused on targeted genetic deletion of Cav1.2 as a strategy to restrict Ca^2+^ influx in pericytes in mice. However, at present, this approach has limited applicability for glaucoma patients. Pharmacological blockers of L-type voltage-gated Ca^2+^ channels such as nifedipine and verapamil, used in the clinic for the management of angina and hypertension, are not pericyte-specific and can have adverse effects (56). AAV vectors, currently used clinically for the treatment of retinal degeneration (57), can be potentially developed to silence Cav1.2 selectively in retinal pericytes of glaucoma patients. Third, we cannot exclude that other paths of Ca^2+^ entry to pericytes, from extracellular or intracellular sources (26), might also play a role in the context of glaucoma, and thus it would be useful to explore their therapeutic potential in future studies. From a translational perspective, the development of strategies to selectively target pericytes and restore Ca^2+^ homeostasis will have wide applications to restore neurovascular health in neurodegenerative diseases while reducing harmful side effects.

## Materials and Methods

### Experimental Animals.

Animal procedures were approved by the University of Montreal Hospital Research Center and followed Canadian Council on Animal Care guidelines. Experiments included adult female and male mice (2 to 6 mo of age, 20 to 35 g) from the following strains: 1) red fluorescent protein under the control of the NG2 (*Cspg4*) promoter (NG2-DsRed) for selective visualization of retinal pericytes (008241; Jackson Laboratory); 2) Ca^2+^ indicator GCaMP6f (fast variant) downstream of the NG2 promoter (NG2-GCaMP6f), generated by crossing NG2-Cre mice (008533; Jackson Laboratory) with Ai95(RCL-GCaMP6f)-D mice (024106; Jackson Laboratory) which harbor the Rosa-CAG-LSL-GCaMP6f::deltaNeo conditional allele with a floxed-STOP cassette; and 3) pericyte-specific conditional deletion of the gene encoding Cav1.2 (*Cacna1c*) generated by crossing NG2-Cre mice (008533; Jackson Laboratory) with floxed *Cacna1c* mice (*Cacna1c^flx/flx^*; 024714; Jackson Laboratory). For two-photon live imaging, we generated albino mice by backcrossing each of these lines with CD-1 mice. Animals were housed in 12-h light–12-h dark cyclic light conditions, with an average in-cage illumination level of 10 lx, and fed ad libitum. All procedures were performed under general anesthesia (20 mg/kg ketamine, 2 mg/kg xylazine, 0.4 mg/kg acepromazine).

### Magnetic Microbead Occlusion Mouse Glaucoma Model.

Unilateral elevation of intraocular pressure was performed by a single injection of magnetic microbeads into the anterior chamber of the mouse eye as described (23). Briefly, we anesthetized the animals and applied a drop of tropicamide on the cornea to induce pupil dilation (Mydriacyl; Alcon). We loaded a custom-made sharpened microneedle attached to a microsyringe pump (World Precision Instruments) with a homogenized magnetic microbead solution (1.5 µL; diameter 4.5 µm, 2.4 × 10^6^ beads; Dynabeads M-450 Epoxy; Thermo Fisher Scientific). Using a micromanipulator, we used the tip of the microneedle to gently puncture the cornea, and injected the microbeads into the anterior chamber avoiding injury to ocular structures such as the lens and iris. A hand-held magnet was used to immediately attract the magnetic microbeads to the iridocorneal angle. Sham controls received an injection of phosphate-buffered saline (PBS). We applied an antibiotic eye drop to the operated eye (Tobrex, tobramycin 0.3%; Alcon) and allowed the animal to recover on a heating pad. We measured the intraocular pressure before and after the procedure, and biweekly thereafter, in awake animals using a calibrated TonoLab rebound tonometer (Icare). For intraocular pressure measurements, a drop of proparacaine hydrochloride (0.5%; Alcon) was applied to the cornea and, holding the tonometer perpendicular to the eye surface, we took and averaged 10 consecutive intraocular pressure readings per eye.

### TPLSM Imaging.

TPLSM live retinal imaging was performed as previously described (15). Mice were anesthetized and placed on a custom-made setup designed to accommodate light stimulation during TPLSM imaging. We kept mice on a homeothermic blanket (Stoelting) to maintain body temperature during imaging (37 °C). We opened the eyelids and used a 6.0 suture, attached to the superior ocular muscle, to gently rotate the eyeball and expose the sclera atop the medial superior and peripheral retina. The conjunctiva over the sclera was gently teased to place a 5-mm-diameter coverslip (Harvard Apparatus) and generate a flat plane for imaging (field of view 400 × 400 μm) using a multiphoton microscope controlled by Zen software (LSM 780; Zeiss). For excitation, we used a mode-locked Ti:sapphire laser (Chameleon Ultra; Coherent) through a water-immersion objective (20×; numerical aperture 1.0; Zeiss). For light-triggered visual stimulation, we generated a flash stimulus (10^2^ cd/m^2^, 6 ms) with a PowerLab unit (ADInstruments) presented using a white light–emitting diode centered relative to the pupil and located 5 mm away from the corneal apex. Stimulus onset (*t* = 0) and TPLSM imaging recording were synchronized offline by identifying the frame at which the light stimulus was registered. We carried out image acquisition using a wavelength of 820 nm to excite TRITC/DsRed or FITC-dextran and a mean laser power at the sample plane of 15 to 50 mW. Imaging was performed throughout the entire thickness of the retina below the sclera (depth 50 to 200 μm) and multiple fields were scanned (25 × 25 μm, 90 × 90 pixels) at 40 Hz and acquired during light stimulation.

### Intravitreal Injections.

We administered the following fluorescent probes or reagents by intravitreal injection (2 µL total volume): TRITC-lectin (5 µg/mL; Sigma), Fluo-4-AM (5 µM; Invitrogen), or AAV serotype 9 carrying GCaMP6f under the control of the synapsin promoter (AAV-GCaMP6f; 1 × 10^13^ particles per milliliter; Addgene). The tip of a custom-made glass micropipette was inserted into the superior quadrant of the eye at an ∼45° angle, through the sclera into the vitreous body, avoiding injury to eye structures or retinal detachment.

### Analysis of Vessel Diameter, IP-TNTs, and Capillary Dynamics.

Pericytes and capillaries in all vascular plexuses and branch orders were included in our in vivo and ex vivo analyses.

#### In vivo.

Immediately prior to TPLSM imaging, we performed tail-vein injection of FITC-coupled dextran (70 kDa, 1 mg/mL in 100 μL; Sigma) or intraperitoneal injection of fluorescein (5% in 100 µL; Novartis Pharma) to label vessels. For live imaging of IP-TNTs, we injected fluorescently tagged lectin (Thermo Fisher Scientific) into the vitreous chamber 1 h prior to imaging. We acquired 40-Hz recordings at a resolution of 512 × 512 pixels (90 × 90 pixels at the region of interest), which were automatically corrected for residual movements with ImageJ (NIH) and the TurboReg plugin (Biomedical Imaging Group). Diameter measurements were then performed by placing a linear probe at the desired location, perpendicular to the fluorescent plane of the filled vessel, using ImageJ (NIH). After projecting the signal every five frames, the fluorescent pattern was exported to a custom R routine freely available (https://www.r-project.org) and the vascular diameter was computed. Recordings with large-amplitude motion, which led to loss of focus during live imaging, were discarded (<5%). To rule out artifacts from movements in the z axis, we imaged the volume of capillaries by capturing five-slice stack series during light stimulation and compared it with single-plane measurements. No vessels were eliminated from our analysis and we recorded thousands of capillaries from all vascular plexuses throughout the retina using an unbiased stereological sampling approach. We normalized diameter changes after light stimulation relative to changes prior to stimulus presentation and classified them as positive or negative based on their dilation or constriction response, respectively. We calculated maximum responses by averaging the global diameter change after a light stimulus. IP-TNT z projections were done with Imaris software (Bitplane).

#### Ex vivo.

For analysis of vessel diameter on fixed flat-mounted NG2-DsRed retinas, we used systematic uniform random sampling as described above. Images of all lectin-labeled microvessels within the three-dimensional (3D) disector frame were acquired with an Axio Imager M2 optical sectioning microscope (40× objective; Zeiss) and analyzed using ImageJ (NIH). The vessel diameter was measured at locations where the circular probe touched the vessel. The number of capillaries within each disector was quantified and capillary density was calculated.

### Blood Flow Measurements.

FITC- or Texas red–coupled dextran (70 kDa, 1 mg/mL in 100 μL; Sigma) was administered by tail-vein injection and blood flow was assessed by quantification of RBCs that crossed a defined location per second before and after light stimulation. RBCs do not take up dextran, and hence they were identified as shadows against the fluorescent background (15). Capillaries from all plexuses of the retina were scanned at 40 Hz and light-evoked blood flow changes were recorded.

### Retinal Immunohistochemistry.

Animals were deeply anesthetized and transcardially perfused with 4% paraformaldehyde (PFA) or methanol. Eyes were immediately collected and processed to generate cryosections as described (58), and then labeled with 488 nm– or 647 nm–coupled lectin (*Bandeiraea simplicifolia*) (5 μg/mL; Thermo Fisher Scientific). We incubated retinal sections with the following primary antibodies: laminin 2 (LAMA2; 2.5 µg/mL; Sigma), Cav1.2 (4 µg/mL; Alomone Laboratories), RBPMS (0.25 µg/mL; PhosphoSolutions), choline acetyltransferase (ChaT; 16.5 µg/mL; Millipore), and SMI-32 (10 µg/mL; BioLegend). We incubated antibodies in blocking solution (10% goat serum albumin in PBS with 0.1% Tween 20) at 4 °C overnight for 3 d, followed by fluorophore-conjugated secondary antibodies (1.5 μg/mL; Jackson ImmunoResearch). Flat-mounted retinas and retinal cross-sections were rinsed and mounted in antifade solution with DAPI (SlowFade; Molecular Probes) for visualization using an epifluorescence microscope (AxioSkop 2 Plus; Zeiss) or a confocal microscope (SP5; Leica Microsystems). Eight retinal cross-sections per eye were analyzed.

### Ca^2+^ Recordings and Quantification.

#### Pericytes and IP-TNTs.

We identified IP-TNTs and their associated pericytes by TPLSM in NG2-GCaMP6f mice after intravitreal injection of TRITC-lectin. Ca^2+^ transients in regions of interest were longitudinally recorded by TPLSM (excitation 920 nm) and signals were calculated as Δ*F/F* = (*F* − *F*_0_)*100/*F*_0_, where *F*_0_ is the fluorescence baseline and *F* is the fluorescence at time *t*. We quantified Ca^2+^ transients automatically using a custom R routine freely available (https://www.r-project.org) when the maximum peak was ≥3 times the SEM over baseline fluorescence, and Ca^2+^ transient frequency was calculated at each region of interest. We defined Ca^2+^ transients as rapid intracellular Ca^2+^ increases in individual pericytes. A subset of Ca^2+^ transients between IP-TNT–connected pericytes was identified and recorded as synchronous Ca^2+^ peaks in linked pericytes within a window of 3 s around each peak, as described (15). To increase the signal-to-noise ratio for detection of low Ca^2+^ levels, we summed the Ca^2+^ signal intensity every two frames (0.4-s lapse), obtained the intensity value of each pixel in every frame using ImageJ (NIH), and color-coded their values over time with a custom R routine (color palette, YlOrRd) freely available (https://www.r-project.org). Fluo-4-AM allowed us to visualize Ca^2+^ levels by combining the signal from 50 frames, but it did not enable the measurement of Ca^2+^ dynamics in pericytes and IP-TNTs. For quantification of Ca^2+^ signals in retinal explants, we superfused the explants from NG2-GCaMP6f mice with oxygenated bicarbonate-buffered artificial cerebrospinal fluid solution (145 mM NaCl, 26 mM NaHCO_3_, 1.2 mM Na_2_HPO_4_, 2.5 mM KCl, 1.3 mM MgCl_2_, 2.5 mM CaCl_2_, 10 mM glucose, pH 7.4) bubbled with 95% O_2_, 5% CO_2_ at 34 °C. Time-lapse images were acquired using a Quorum Technologies confocal microscope with a CSU10B (Yokogawa) spinning head mounted on a BX61W1 fluorescence microscope (Olympus) and connected to an ORCA-ER camera (Hamamatsu Photonics). Images were captured using Volocity software (Improvision) and analyzed by ImageJ (NIH). For ex vivo quantification of Ca^2+^ signals, we removed the eyes, fixed them in 4% PFA, and flat-mounted the retinas. Using an unbiased stereological sampling approach, we acquired images over the entire retina with identical exposure times and gain settings for all experimental and control groups (40× objective; ApoTome 2; Zeiss). Raw fluorescence intensity in each cell, acquired from images obtained using the same exposure time for all cohorts, was measured manually with ImageJ (NIH). Background fluorescence obtained from three surrounding square areas was subtracted to yield the final value for each region of interest. High-Ca^2+^ pericytes were defined as pericytes with an intensity ≥2 times the SEM over sham pericyte fluorescence. Ca^2+^ signals in *Cacna1c^−/−^* and wild-type littermate mice were visualized by intravitreal injection of the Ca^2+^ indicator Fluo-4-AM (Invitrogen) 1 h prior to imaging.

#### RGCs.

To evaluate Ca^2+^ responses in RGCs, we administered AAV-GCaMP6f by intravitreal injection 3 wk prior to TPLSM imaging. RGCs expressing GCaMP6f were scanned at 12 Hz and Ca^2+^ signals were analyzed in circular regions of interest encompassing the entire soma. We examined light-evoked Ca^2+^ responses by averaging the fluorescence intensity of all pixels within the region of interest using ImageJ (NIH) after background subtraction. We calculated the Δ*F/F* peak and decay time, defined as the time to fall to one-third of the Δ*F/F* peak, with a custom R routine freely available (https://www.r-project.org), and light-responsive neurons were defined as cells with Δ*F/F* >2.

### Quantification of RGCs and IP-TNTs.

For RGC quantification, whole retinas were labeled with an anti-RBPMS antibody as described above, mounted with the nerve fiber layer side up, and visualized with an Axio Observer (Zeiss). IP-TNTs were visualized in flat-mounted retinas labeled with fluorescently tagged lectin (Thermo Fisher Scientific). Retinal images were obtained using an Axio Imager M2 optical sectioning microscope (20× objective; Zeiss) equipped with an automatically controlled specimen stage for *x*-, *y*-, and *z*-axis movement, a color camera (Axiocam 509 mono; Zeiss), and image analysis software (Zen; Zeiss). Using the stereological random sampling method described above, RBPMS-labeled RGCs and lectin-positive IP-TNTs were counted using 3D disectors (stacks) throughout the entire retina, and the number of RGCs or IP-TNTs was calculated (15, 59).

### Statistical Analysis.

We always carried out data analysis blinded by third-party concealment of treatment using uniquely coded samples. The number of animals used in each experiment as well as the number of cells or structures analyzed are indicated in the figure legends. All values are provided as the mean ± SEM, and individual values are presented in each graph. Statistical analysis was performed with Prism 9 (GraphPad). We evaluated all cohorts with normality (Shapiro–Wilk) and variance (*F*) tests. We compared values corresponding to vessel diameter, blood flow, probability of capillary blockage, Ca^2+^ signal amplitude, Ca^2+^ decay, number of IP-TNTs, RGC density, and Ca^2+^ transients. Stereological quantifications were compared by means of two-tailed Student’s *t* or Mann–Whitney *U* tests, where appropriate. For multiple comparisons, we used ANOVA followed by Dunnett’s, Tukey’s, or Kruskal–Wallis tests, where appropriate. A *P* value ≤ 0.05 was considered significant. All regression lines of diameter-change graphs were fitted with the same order between experimental and control cohorts.

## Supplementary Material

Supplementary File

Supplementary File

Supplementary File

Supplementary File

Supplementary File

Supplementary File

Supplementary File

Supplementary File

Supplementary File

## Data Availability

All the data analyzed in this study, including raw data, are included in the article and/or supporting information.
