# Supplementary material for: Pericyte dysfunction and loss of interpericyte tunneling nanotubes promote neurovascular deficits in glaucoma
Source: Proc Natl Acad Sci U S A. 2022 Feb 8;119(7):e2110329119. doi: 10.1073/pnas.2110329119 (PMC8851476; doi:10.1073/pnas.2110329119)
Supplement: Supplementary File [file pnas.2110329119.sapp.pdf]

PNAS
www.pnas.org

Supplementary Information for

Pericyte dysfunction and loss of inter-pericyte tunneling nanotubes promote neurovascular deficits in glaucoma

Luis Alarcon-Martinez [a,b,*,†], Yukihiro Shiga [a,b,†], Deborah Villafranca-Baughman [a,b], Nicolas Belforte [a,b], Heberto Quintero [a,b], Florence Dotigny [a,b], Jorge L. Cueva Vargas [a,b], Adriana Di Polo [a,b,*].

† These authors contributed equally to this work.

*Corresponding authors: adriana.di.polo@umontreal.ca, lalarcon@um.es.

a. Department of Neuroscience, Université de Montréal, PO box 6128, Station centre-ville, Montreal, Quebec H3C 3J7, Canada.
b. Neuroscience Division, Centre de recherche du Centre Hospitalier de l'Université de Montréal (CRCHUM), 900 Saint Denis Street, Montreal, Quebec H2X 0A9, Canada.

This PDF file includes:

Figures S1 to S5
Table 1
Legends for Movies S1 to S8
Supporting Information for Movie S7

Other supplementary materials for this manuscript include the following:

Movies S1 to S8

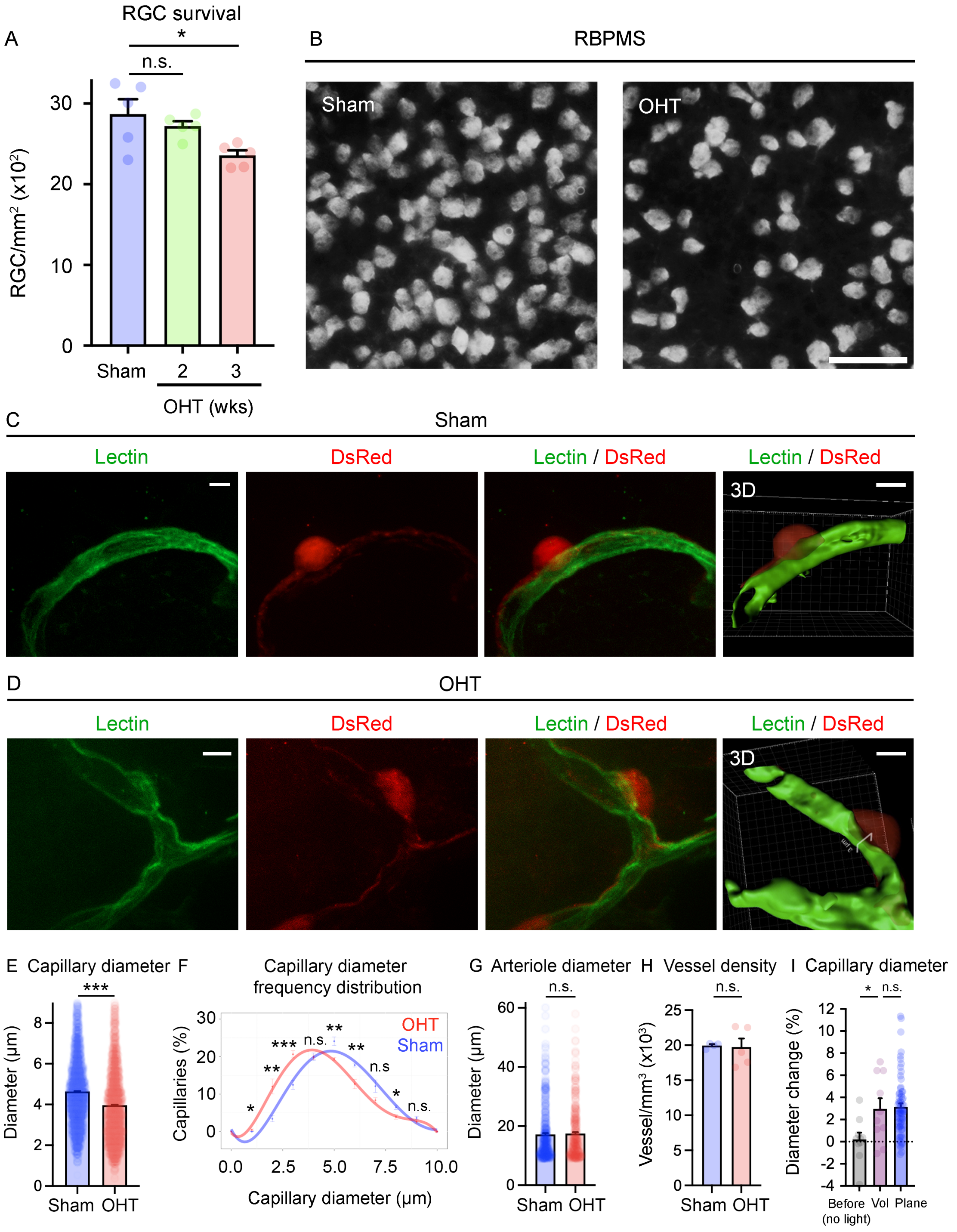

Fig. S1. RGC death and capillary dysfunction in a mouse occlusion glaucoma model. (A) Intracameral injection of magnetic microbeads results in ocular hypertension and statistically significant RGC loss at 3 weeks after this procedure (sham: N=5 mice; OHT-2wks: N=5 mice; OHT-3wks: N=5 mice; two-tailed ANOVA Tukey's test, *P<0.05, n.s.: not significant). (**B**) Representative

images of flat-mounted retinas labeled with the RGC-specific marker RBPMS showing significant RGC death at 3 weeks after glaucoma induction. (**C, D**) Representative images of a retinal capillary and its associated pericyte in a sham (**C**) and glaucomatous retina (**D**, OHT-3wk). The rightmost panel is a 3D reconstruction of each pericyte/capillary pair to quantify vessel diameter at pericyte locations. (**E**) OHT leads to a decrease in capillary diameter at pericyte locations (sham: n=3824 capillaries, N=5 mice; OHT: n=3668 capillaries, N=5 mice; Mann-Whitney U test, ***P<0.001). (**F**) Frequency distribution of changes in capillary diameter shows a left shift in the curve for glaucomatous retinas (red trace) relative to sham controls (blue trace) indicating a higher number of capillaries with reduced diameter during OHT (sham: N=5 mice; OHT: N=5, two-tailed Student's t-test, ***P<0.001, **P<0.01, *P<0.05, n.s.: not significant). (**G**) The diameter of arterioles did not change in glaucoma indicating that the observed responses were specific to capillaries (sham: n=442 arterioles, N=5 mice; OHT: n=351 arterioles, N=5 mice; Mann-Whitney U test, n.s.: not significant). (**H**) Capillary density was not altered in glaucoma (sham: N=4 mice, OHT-2wks: N=5 mice, two-tailed Student's t-test, n.s.: not significant). (**I**) Maximum response graph obtained from capillary volume or single-focal plane analysis confirmed that light-triggered capillary reactivity is similar in both cases supporting that light-evoked capillary diameter changes are not due to artefactual movements in the z axis (before (no light): plane measurements, n=10 capillaries, N=2 mice; volume measurements: n=10 capillaries, N=2 mice; plane measurements: n=70 capillaries, N=5 mice; two-tailed ANOVA Dunnett's test, *P<0.05, n.s.: not significant). Data are presented as mean values +/- S.E.M. Scale bars: 50 μm (A), 5 μm (C, D).

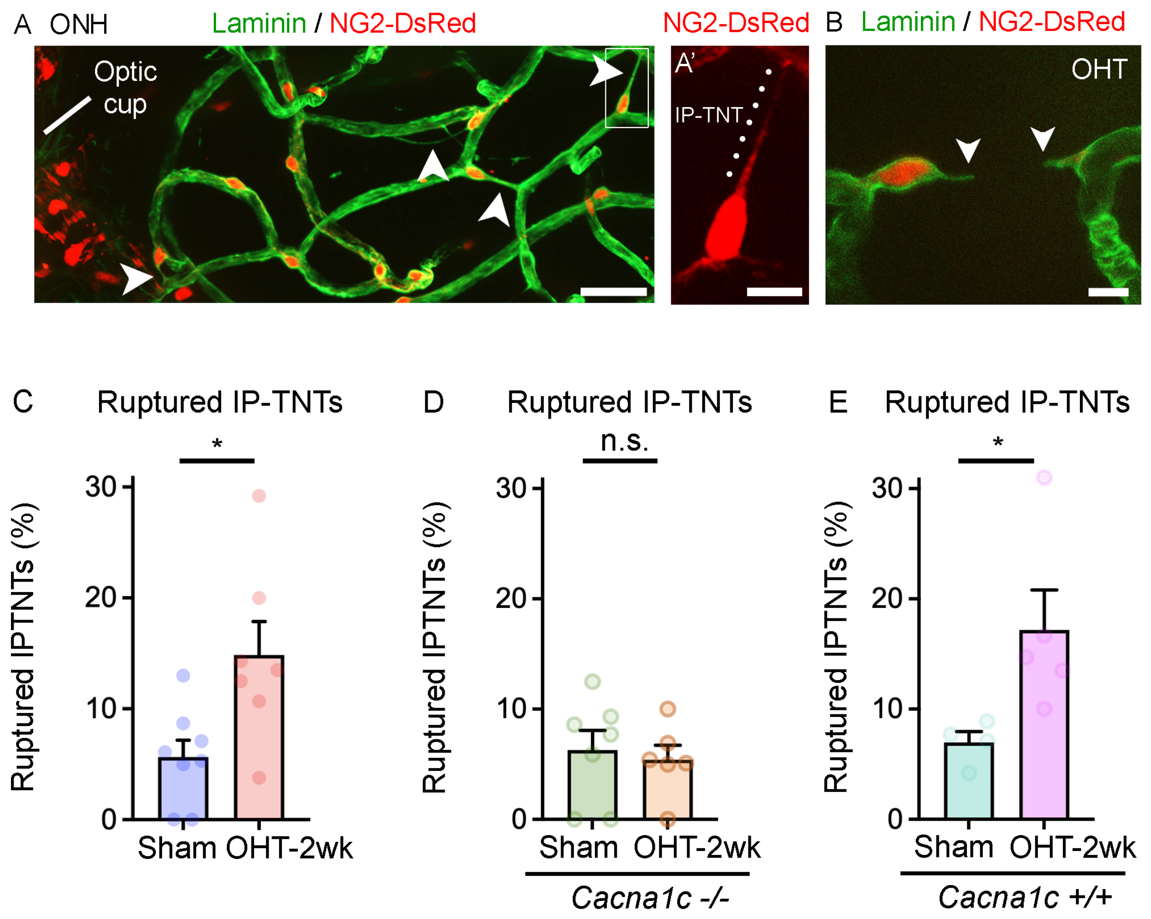

Fig. S2. Characterization of IP-TNTs in the glaucomatous optic nerve head. (**A**) Analysis of the optic nerve head (ONH) in an NG2-DsRed mouse shows a network of IP-TNTs, labeled with laminin, connecting pericytes between capillaries. Endogenous DsRed fluorescence is shown in higher magnification inset (A'). (**B, C**) Similar to the retina, high intraocular pressure leads to IP-TNT rupture in the ONH (sham: n= 185 IP-TNTs, N=8 mice; OHT-2wks: n= 152 IP-TNTs, N=7 mice; two-tailed Student's t-test, *P<0.05). (**D, E**) Pericyte-specific deletion of *Cacna1c* protects IP-TNTs (panel **D**, sham: n=191 IP-TNTs, N=7 mice; OHT-2wks: n=183 IP-TNTs, N=6 mice; two-tailed Student's t-test, n.s.: not significant), whereas significant IP-TNT damage occurs in wildtype littermate controls (panel **E**, sham: n=150 IP-TNTs, N=4 mice; OHT-2wks: n=167 IP-TNTs, N=5 mice; two-tailed Student's t-test, *P<0.05). Data are presented as mean values +/- S.E.M. Scale bars: 20 µm (A), 5 µm (A', B).

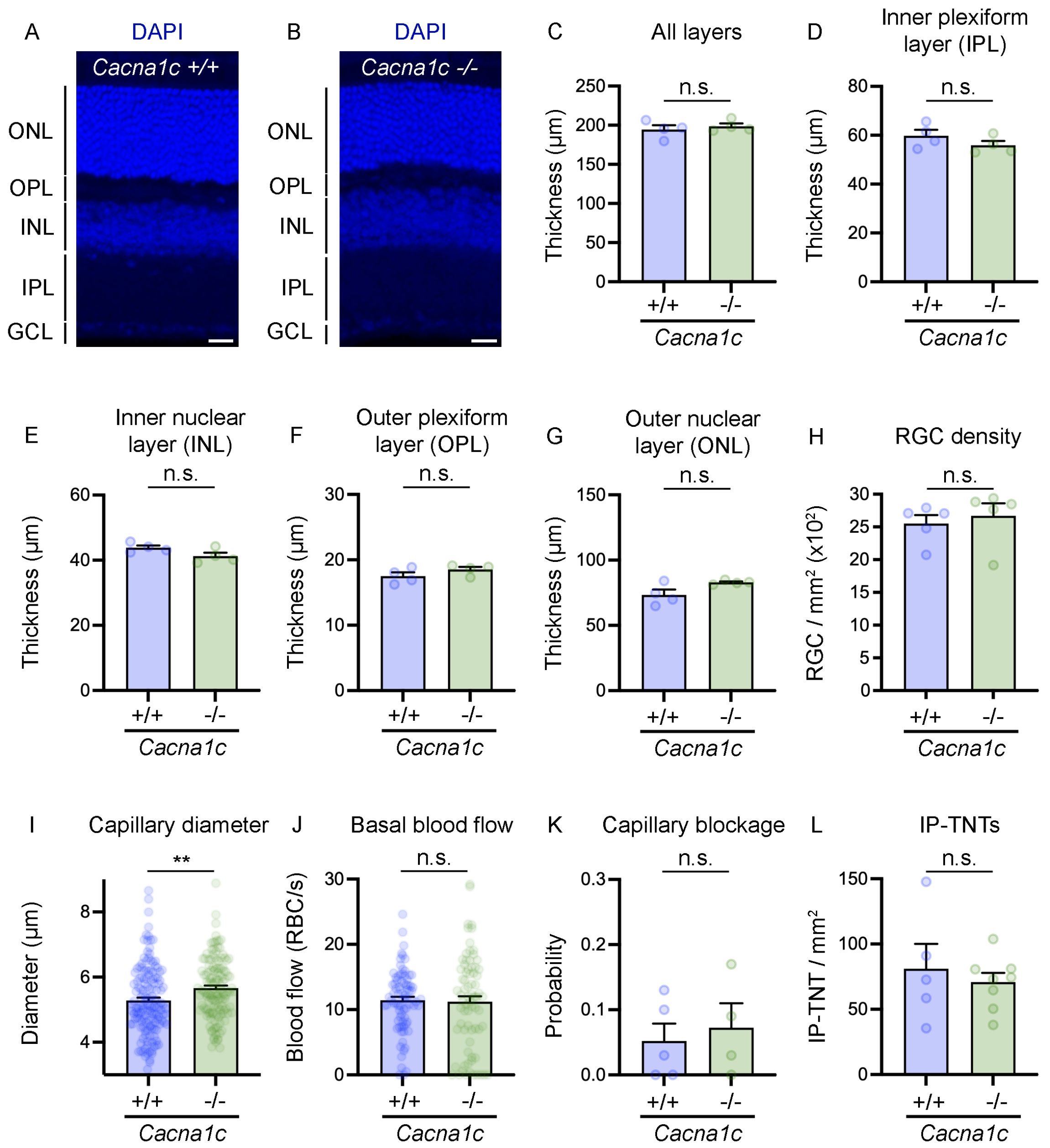

Fig. S3. Cav1.2 depletion does not cause major alterations in *Cacna1c* null retinas. (**A, B**) Representative images of DAPI-stained retinal cross sections from *Cacna1c* null mice and wildtype littermate controls show no major alterations in tissue organization (ONL: outer nuclear layer, OPL: outer plexiform layer, INL: inner nuclear layer, IPL: inner plexiform layer, GCL: ganglion cell layer). (**C-G**) Quantitative analysis confirms similar retinal layer thickness between *Cacna1c*$^{-/-}$ and *Cacna1c*$^{+/+}$ mice ruling out major retinal abnormalities (*Cacna1c*$^{+/+}$: N=4 mice; *Cacna1c*$^{-/-}$: N=4 mice; two-tailed Student's t-test, n.s.: not significant). (**H**) Quantification of RGC density also shows

no significant differences between *Cacna1c*$^{-/-}$ and *Cacna1c*$^{+/+}$ retinas (*Cacna1c*$^{+/+}$: N=5 mice; *Cacna1c*$^{-/-}$: N=5 mice; two-tailed Student's t-test, n.s.: not significant). (**I**) Basal capillary diameter is slightly increased in non-injured sham-operated *Cacna1c*$^{-/-}$ relative to *Cacna1c*$^{+/+}$ mice (*Cacna1c*$^{+/+}$: n=147 capillaries, N=5 mice; *Cacna1c*$^{-/-}$: n=135 capillaries, N=5 mice; two-tailed Student's t-test, **P<0.01). (**J-L**) Basal blood flow, capillary blockage probability, and IP-TNT numbers are similar between these groups confirming the absence of major vascular alterations (panel **J**, *Cacna1c*$^{+/+}$: n=81 capillaries, N=5 mice; *Cacna1c*$^{-/-}$: n=75 capillaries, N=4 mice; two-tailed Student's t-test, n.s.: not significant; panel **K**, *Cacna1c*$^{+/+}$: n=81 capillaries, N=5 mice; *Cacna1c*$^{-/-}$: n=75 capillaries, N=4 mice; two-tailed Student's t-test, n.s.: not significant, panel **L,** *Cacna1c*$^{+/+}$: N=5 mice; *Cacna1c*$^{-/-}$: N=8 mice; unpaired Student's t-test, n.s.: not significant). Data are presented as mean values +/- S.E.M. Scale bars: 20 μm.

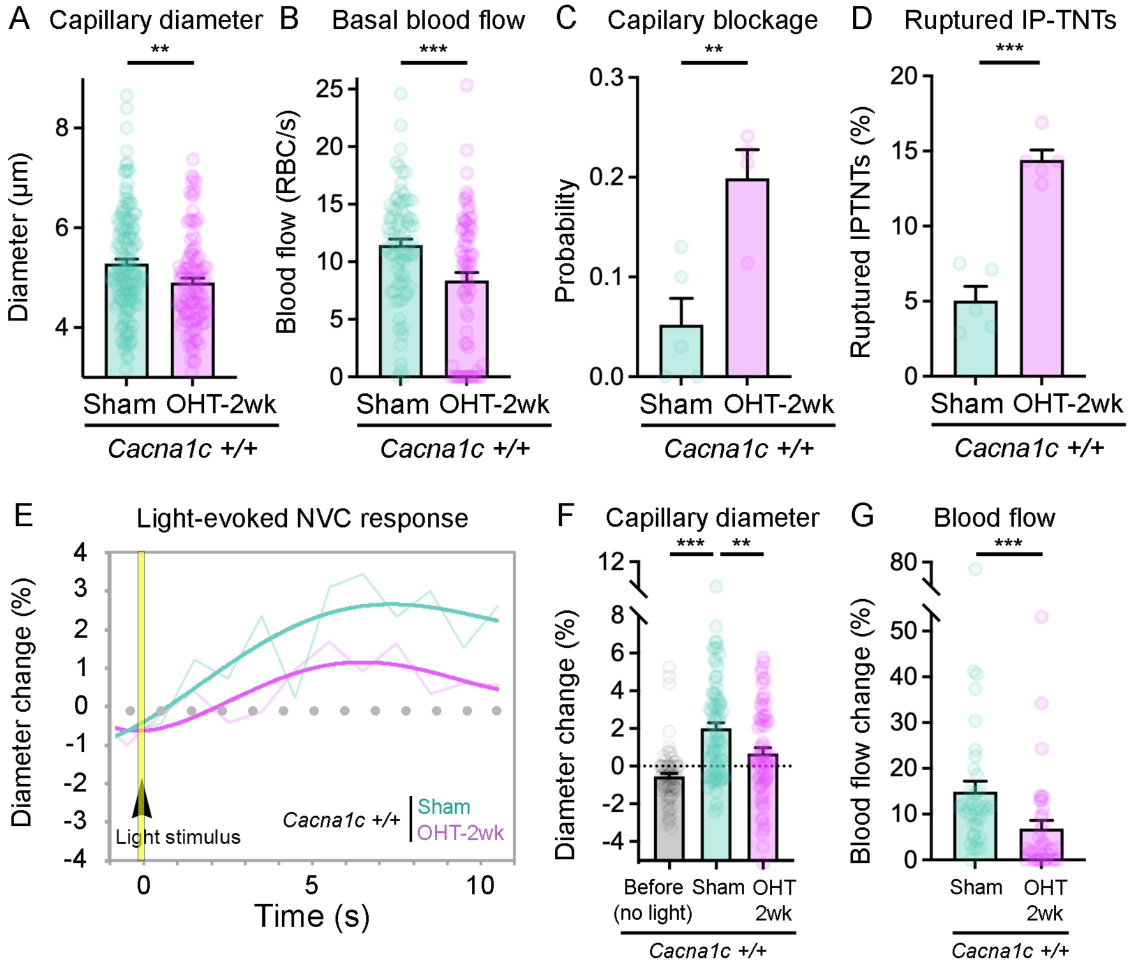

Fig. S4. *Cacna1c* wild-type littermate controls present ocular hypertension-induced capillary and neurovascular dysfunction. (A-D) Analysis of vascular function in *Cacna1c*$^{+/+}$ mice subjected to glaucoma demonstrates major alterations relative to sham-operated controls including reduced capillary diameter (panel **A**, sham: n=147 capillaries, N=5 mice; OHT-2wks: n=109 capillaries, N=4 mice; Mann-Whitney U test, **P<0.01), decreased basal blood flow (panel **B,** sham: n=81 capillaries, N=5 mice; OHT-2wks: n=75 capillaries, N=4 mice; two-tailed Student's t-test, ***P<0.001), capillary blockage (panel **C,** sham: n=81 capillaries, N=5 mice; OHT-2wks: n=75 capillaries, N=4 mice; two-tailed Student's t-test, **P<0.01), and IP-TNT rupture (panel **D,** sham: n=1194 IP-TNTs, N=5 mice; OHT-2wks: n=1119 IP-TNTs, N=5 mice; two-tailed Student's t-test, ***P<0.001). (**E**) Longitudinal TPLSM-recorded light-evoked neurovascular responses show impaired capillary vasodilation in *Cacna1c*$^{+/+}$ mice subjected to glaucoma (sham: n=78 capillaries, N=5 mice; OHT: n=67 capillaries, N=4 mice). (**F, G**) Maximum response graphs show impaired

light-evoked capillary reactivity (panel **F**, before (no light) sham: n=78 capillaries, N=5 mice; sham: n=78 capillaries, N=5 mice; OHT-2wks: n=67 capillaries, N=4 mice; two-tailed ANOVA Tukey's test, **P<0.01, ***P<0.001) and reduced blood flow (panel **G,** sham: n=38 capillaries, N=5 mice; OHT-2wks: n=37 capillaries, N=4 mice; Mann-Whitney U test, ***P<0.001) in glaucomatous *Cacna1c*$^{+/+}$ mice relative to sham controls.

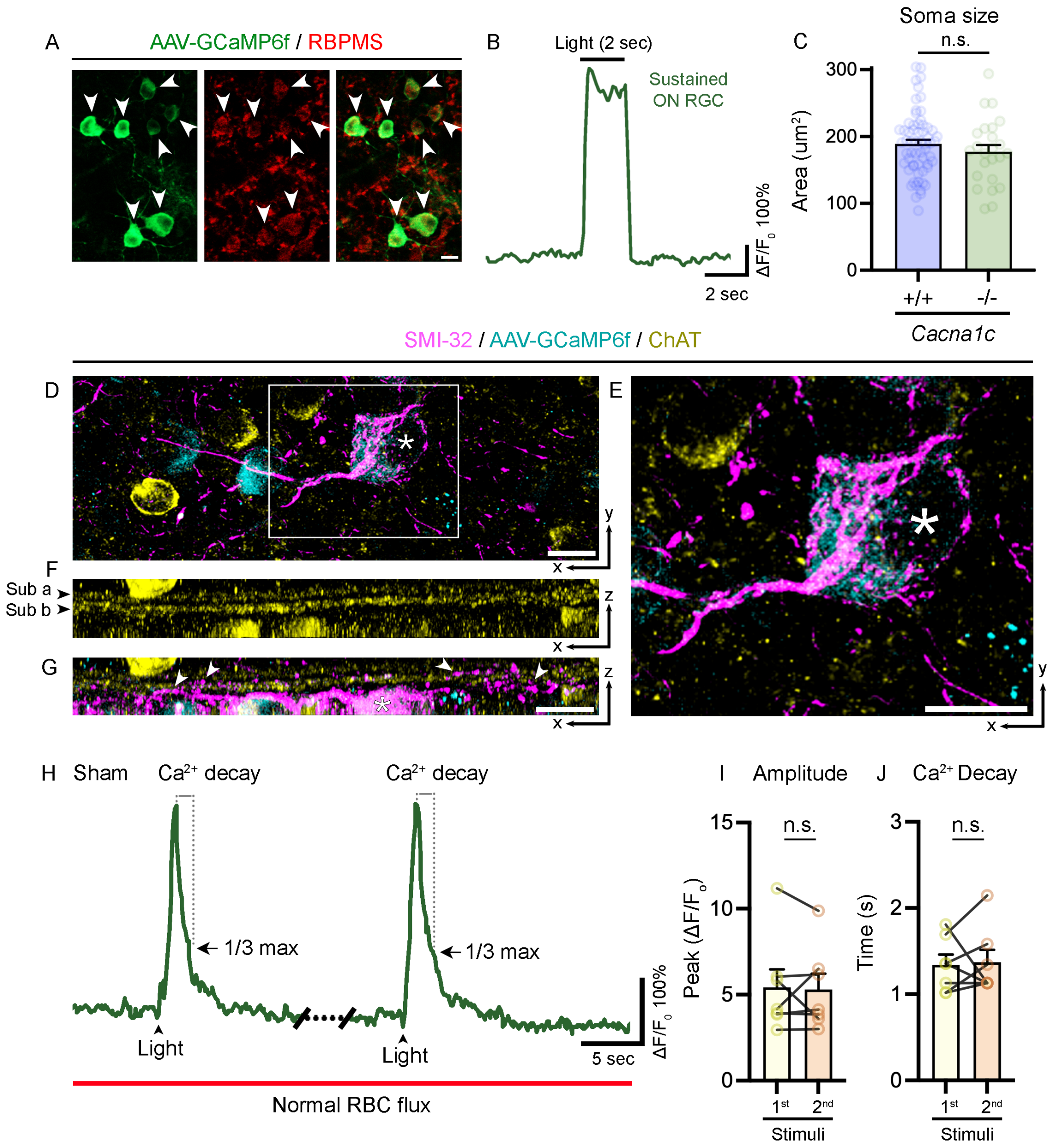

Fig. S5. Characterization of αON-S RGC targeted for Ca^{2+} dynamics recordings. (**A**) Representative image of a flat-mounted retina shows expression of GCaMP6f (green) in RBPMS-positive RGC (red, arrowheads) at three weeks after AAV-GCaMP6f administration. (**B**) Representative trace of a αON-S RGC response following a 2-sec flash light stimulus. (**C**) Analysis of RGC soma area is consistent with αON-S cells, and demonstrates that Cav1.2 depletion does not alter the surface area of the recorded neurons (*Cacna1c*$^{+/+}$: n=61 neurons, N=11 mice;

Cacna1c$^{-/-}$: n=24 neurons, N=6 mice; two-tailed Student's t-test, n.s.: not significant). (**D, E**) Immunohistochemical *post-hoc* analysis of neurofilament H protein (SMI-32, pink) and GCaMP6f (green) confirms the identity of αON-S RGC (asterisk). (**F, G**) ChAT labeling corroborates αON-S RGC dendritic stratification in the proximal sublamina b (arrowheads in **G**). (**H**) Light-evoked Ca^{2+} dynamics of an RGC fed by a capillary with normal RBC flux, using two recurrent light stimuli, show preservation of the response pattern. (**I, J**) The use of two light stimuli did not alter the Ca^{2+} response amplitude (panel **I,** 1st stimulus: n=7 neurons, N=3 mice; 2nd stimulus: n=7 neurons, N=3 mice; two-tailed paired Student's t-test, n.s.: not significant) or the Ca^{2+} decay time (panel **J,** 1st stimulus: n=7 neurons, N=3 mice; 2nd stimulus: n=7 neurons, N=3 mice; two-tailed paired Student's t-test, n.s.: not significant). Scale bar: 10 μm (A, D-G).

Table 1. Intraocular pressure elevation in experimental and control groups.

Time (wks)	Sham-operated eyes			Microbead-injected eyes			Sham vs. injected eyes
	Mean	SEM	N	Mean	SEM	N	P-value (Two-tailed Student's t-test)
0	13.1	0.3	25	13.2	0.3	44	0.3526
1	15.5	0.5	6	20.0	0.8	16	0.0034
2	13.6	0.5	25	22.0	0.8	20	<0.0001
3	12.6	0.8	16	21.8	0.6	20	<0.0001

Movie S1 (separate file). TPLSM blood flow quantification in sham retinas. Blood flow visualized by TPLSM in sham-operated retinas allows the quantification of red blood cells (RBC) crossing a pre-determined location per second. RBC do not take up tail vein-injected dyes, hence are visualized as shadows against the fluorescent plasma. The counter (red) shows the number of RBC flowing through a single capillary. Scale bar = 5 µm. Temporal resolution: 25 frames per second (fps).

Movie S2 (separate file). TPLSM blood flow quantification in glaucomatous retinas (OHT- 2 weeks). Blood flow visualized by TPLSM in glaucomatous retinas (OHT-2wks) show decreased single capillary blood flow (RBC/sec) relative to sham-operated control mice (Movie S1). The counter (red) shows the number of RBC flowing through a single capillary. Scale bar = 5 µm. Temporal resolution: 40 fps.

Movie S3 (separate file). TPLSM blood flow quantification in glaucomatous retinas (OHT- 3 weeks). Blood flow visualized by TPLSM in glaucomatous retinas (OHT-3wks) show decreased single capillary blood flow (RBC/sec) relative to sham-operated control mice (Movie S1). The counter (red) shows the number of RBC flowing through a single capillary. Scale bar = 5 µm. Temporal resolution: 40 fps.

Movie S4 (separate file). Pericyte-mediated capillary constriction in glaucoma. TPLSM live imaging of a glaucomatous NG2-DsRed retina shows a pericyte (red, arrow) constricting a capillary (green) causing RBC to stall (arrowhead) thus interrupting blood flow. Blood flow in the upstream arteriole was not affected by pericyte-induced capillary constriction. Scale bar = 10 µm. Temporal resolution: 0.5 fps.

Movie S5 (separate file). TPLSM live imaging of Ca^{2+} transients in IP-TNT-coupled pericytes. TPLSM live imaging of NG2-GCaMP6f retinas shows Ca^{2+} transients, visualized in green, in a proximal pericyte (1) and a distal pericyte process (dpp, 2) linked by an IP-TNT labeled with lectin

(red). Ca^{2+} transients are measured as spontaneous changes in IP-TNT-coupled pericytes. Scale bar = 5 µm. Temporal resolution: 2.5 fps.

Movie S6 (separate file). *Ex vivo* recordings of Ca^{2+} transients in IP-TNT-coupled pericytes. A retinal explant from an NG2-GCaMP6f mouse shows the soma of two IP-TNT-coupled pericytes labeled with lectin (red). Ca^{2+} transients are visualized in proximal (1) and distal (2) pericytes linked by an IP-TNT (green). Scale bar: 5 µm. Temporal resolution: 1 fps.

Movie S7 (separate file). Ca^{2+} transients between IP-TNT-coupled pericytes. (**A**) Ca^{2+} transients are detected in a proximal pericyte (left), distal pericyte (right), and interconnecting IP-TNT (dotted line) in a NG2-GCaMP6f mouse retina. Ca^{2+} transients are visualized in green, the white arrowhead shows peaks of Ca^{2+} changes, and each structure is visualized with lectin (red). Bottom traces show normalized Ca^{2+} transients at each region of interest (proximal pericyte, IP-TNT, and distal pericyte). Scale bars: 5 µm. Temporal resolution: 5 fps. (**B**) Additional analysis of Ca^{2+} signals is shown at the end of the movie. Ca^{2+} signal intensity was summed every two frames to obtain the intensity value of each pixel in every frame (see Methods). Color-coded Ca^{2+} intensity over time in Movie S7 shows Ca^{2+} transients in IP-TNT-linked pericytes. Scale bar: 5 µm.

Movie S8 (separate file). Simultaneous live TPLSM imaging of light-evoked RGC responses and single capillary blood flow in glaucomatous retinas. Longitudinal recordings of light-evoked single-RGC Ca^{2+} responses as well as blood flow in the capillary serving the same neuron before and after pericyte-induced vessel constriction during glaucoma. In conditions of normal capillary blood flow, RGC Ca^{2+} responses are robust and decayed rapidly, but when blood flow is compromised (arrowhead shows trapped RBC) signal amplitude is reduced and Ca^{2+} decay is delayed. Scale bar: 5 µm. Temporal resolution: 10 fps.

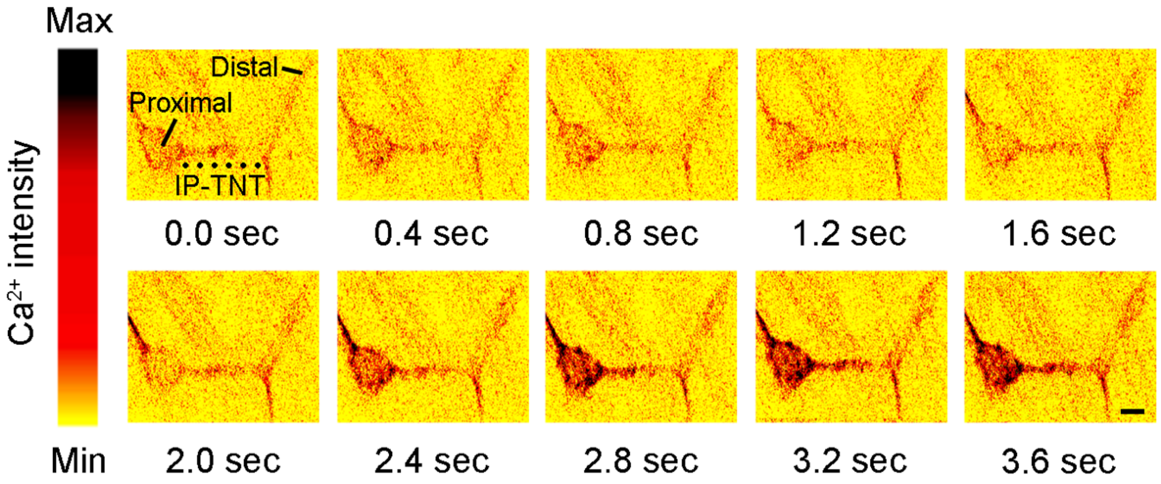

Supporting Information for Movie S7. Color-coded Ca2+ intensity over time in Movie S7 shows Ca2+ transients in IP-TNT-linked pericytes. Scale bar: 5 μm.
